# Two countries, similar practices: the political practices of the food industry influencing the adoption of key public health nutrition policies in Guatemala and Panama

**DOI:** 10.1017/S1368980022001811

**Published:** 2022-11

**Authors:** Maria F Kroker-Lobos, Lourdes Analí Morales, Manuel Ramírez-Zea, Stefanie Vandevijvere, Beatriz Champagne, Melissa Mialon

**Affiliations:** 1 INCAP Research Center for the Prevention of Chronic Diseases, Institute of Nutrition of Central America and Panama, Guatemala City, Guatemala; 2 Sciensano, Ixelles, Belgium; 3 Coalition for Americas’ Health, CLAS, Dallas, TX, USA; 4 Trinity College Dublin, Luce Hall, Pease St, D02 H308 Dublin, Ireland

**Keywords:** Corporate Political Activity, Guatemala, Panama, Food industry, Commercial determinants of health

## Abstract

**Objective:**

To identify the corporate political activity (CPA) strategies used by food industry actors during the development of two public health nutrition policies in Central America: Law #570 (taxation of sugar-sweetened beverages) in Panama and Bill #5504 (labelling and food marketing regulations) in Guatemala.

**Design:**

We triangulated data from publicly available information from 2018 to 2020, (e.g. industry and government materials; social media material) with semi-structured interviews with key stakeholders.

**Setting:**

Guatemala and Panama.

**Participants:**

Government, academia and international organisations workers in health and nutrition.

**Design:**

CPA strategies were categorised according to an existing internationally used taxonomy into action-based, instrumental strategies (coalition management, information management, direct involvement and influence in policy, legal action) and discursive strategies.

**Results:**

Instrumental strategies included the establishment of relationships with policymakers and direct lobbying against the proposed public policies. Discursive strategies were mainly criticising on the unfounded ground that they lacked evidence of effectiveness and will imply negative impacts on the economy. The industry pointed at individuals for making their own food choices, in order to shift the focus away from the role of its products in contributing to ill health.

**Conclusion:**

We provide evidence of the political practices used by the food industry to interfere with the development and implementation of public health nutrition policies to improve diets in Central America. Policymakers, public health advocates and the public should be informed about those practices and develop counterstrategies and arguments to protect the public and policies from the vested interests of the food industry.

Diets in Central America are characterised by an increasing consumption of ultra-processed food and drink products, including sugar-sweetened beverages (SSB)^(1,2)^. That consumption is one of the main risk factors for obesity and diet-related non-communicable diseases (NCD), the leading causes of disability and premature mortality in the region^(3)^.

Evidence from meta-analyses shows that the consumption of ultra-processed food products with excessive amounts of energy and critical nutrients such as fats, saturated fats, trans fats, sodium and added sugars is associated with an increased risk of developing obesity, diabetes, cardiovascular diseases, cerebrovascular diseases, cancers, depression and increases all-cause mortality in adults^(4–9)^. In children, the consumption of ultra-proccessed products worsens their diets and health by displacing breastfeeding, fruits and vegetables consumption, increasing their intake of saturated fats, sugars and sodium intake, and their blood lipid levels and body fat^(10–13)^.

In 2014, the Central American Commission of Ministries of Health approved the implementation of the Strategy for Childhood Obesity Prevention for Central America, in which cost-effective, internationally recommended policy interventions were proposed, such as the implementation of an interpretive nutrition front-of-pack labelling (FOPL) system, limits to the marketing of unhealthy food products targeted at children and SSB taxation^(14)^. Currently, two countries in the Central American region have recently proposed or approved two of these law initiatives, as detailed next.

In 2018, the Parliamentary Front against Hunger in Guatemala submitted the Promotion of Healthy Eating Law initiative (Bill #5504) to the Congress, aiming to introduce a new nutrition FOPL system and regulate marketing targeted at children and adolescents under 18^(15)^. Two commissions within the Congress have positioned themselves in favour of the initiative; however, the debate process is still waiting, as of May 2022.

In 2019, the National Assembly of Panama approved the Law #570 to implement a tax on SSB of 7 % for sodas; 10 % for syrups and concentrates for SSB production and 5 % for the rest of SSB^(16)^. Panama’s food industry publicly asked to the President of the Republic that he partially vetoed that Law, and consequently, its implementation was delayed for a period of time^(17)^.

There is increasing evidence that the slow adoption and implementation of public health nutrition policies in Latin America and elsewhere is indeed due, to a large extent, to the strong opposition from the food industry^(18–20)^. A pilot study commissioned by the Pan American Health Organization in 2018 showed that food industry actors are using different corporate political activity (CPA) strategies in Latin America to influence public health policy, research and practice^(21)^. CPA is defined as ‘corporate attempts to shape government policy in ways favorable to the industry’^(22)^.

CPA strategies are commonly classified, using an internationally developed and implemented taxonomy, as: (1) coalition management, (2) information management, (3) direct involvement and influence in policy, (4) legal action and (5) discursive strategies^(22)^. A detailed description of these instrumental and discursive strategies is presented in Tables 1 and 2, respectively.

**Table 1:** Corporate political activity of the food industry: instrumental strategies, adapted from Mialon et al.^(13)^, based on the findings of the current study

Instrumental Strategies	Practices	Mechanism
Coalition management	Establish relationships with key opinion leaders and health organisations	Promote public–private interactions with health organisations Support professional organisations, including through funding and/or advertising in their publications Establish informal relationships with key opinion leaders Support the placement of industry-friendly personnel within health organisations
	Seek involvement in the community	Undertake corporate philanthropy Support physical activity initiatives Support events (such as for youth or the arts) and community-level initiatives
	Establish relationships with the media	Establish close relationships with media organisations, journalists and bloggers to facilitate media advocacy
	Constituency fabrication	Establish fake grassroots organisations (‘astroturfing’) Procure the support of community and business groups to oppose public health measures
	Opposition fragmentation and destabilisation	Discredit public health advocates personally and publicly, e.g. through the media, blogs Infiltrate, monitor the operation and advocacy strategies of public health advocates, groups and organisations Create antagonism between professionals
Information management	Production	Fund research, including through academics, ghost writers, own research institutions and front groups
	Amplification	Cherry-pick data that favours the industry, including use of non-peer-reviewed or unpublished evidence Participate in and host scientific events Propose industry-sponsored education
	Suppression	Suppress the dissemination of research that does not fit the industry’s interests Emphasise disagreement among scientists and focus on doubt in science Criticise evidence and emphasise its complexity and uncertainty
	Credibility	Fronting: concealing industry links to information/evidence, including through the use of scientists as advisers, consultants or spokespersons
Direct involvement and influence in policy	Indirect access	Lobby directly and indirectly (through third parties) to influence legislation and regulation so that it is favourable to the industry Use the ‘revolving door’, i.e. ex-food industry staff work in government organisations and vice versa
	Incentives	Fund and provide financial incentives to political parties and policymakers (donations, gifts, entertainment or other financial inducements)
	Threats	Threaten to withdraw investments if new public health policies are introduced Intimidation
	Actors in government decision making	Seek involvement in working groups, technical groups and advisory groups Provide technical support and advice to policymakers (including consultation)
	Development and promotion of private-public initiative and self-regulation*	Position against regulations such: ‘we respect the law, so we don’t need any new law’* Direct opposition to regulation or positions promoting self-regulation^α^
Legal actions	Use legal action (or the threat thereof) against public policies or opponents	Litigate or threaten to litigate against governments, organisations or individuals
	Influence the development of trade and investment agreements	Influence the development of trade and investment agreements such that clauses favourable to the industry are included (e.g. limited trade restrictions, mechanisms for corporations to sue governments)

* New corporate political activity practices and mechanisms are identified in the present study.

**Table 2:** Corporate political activity of the food industry: discursive strategies, adapted from Mialon et al.^(13)^, based on the findings of the current study^(22)^

Discursive Strategies	Domain	Argument
Discursive strategies	The Economy	Stress the number of jobs supported and the money generated for the economy
	Governance	Demonise the ‘nanny state’
	Good Governance*	Industry suggesting how government should proceed*
	Expected food industry costs	Policy will lead to reduced sales/jobs Cost of compliance will be high Implementation will promote contraband*
	Frame the debate on diet- and public health-related issues	Stress the good traits of the food industry Shift the blame away from the food industry and its products, e.g. focus on individual responsibility, role of parents, physical inactivity Promote industry´s preferred solutions: education, balanced diets, information, public private initiatives, self-regulation (reformulation

* New corporate political activity practices and/or mechanisms identified in the present study.

Examples of CPA strategies include, for example: lobbying, where the industry promotes and/or defends its interests amongst policymakers; the promotion of deregulation; donations to political parties and the establishment of relationships with the media, amongst other third parties^(23)^. Even if there is opposition by the food industry against both Law #570 in Panama and Bill #5504 in Guatemala, as discussed above^(17,24)^ and as experienced first hand by the authors of the present paper, little is however known about the broad range and extent of CPA strategies used by the food industry in those cases.

Hence, the aim of our study was to identify the CPA strategies used by the food industry during the recent development of those two public health nutrition policies in Guatemala and Panama.

## Methods

Our study consisted of a mapping of the CPA of the food industry, using a research protocol developed by INFORMAS^(23)^, which has been successfully implemented in more than twenty countries to date (see for example^(19,21,25)^). The INFORMAS protocol proposes to use publicly available information across different sources, including industry and government material. The availability of publicly available information is limited in Central America; hence, we triangulated that data with semi-structured interviews with key stakeholders in health and nutrition, such as policymakers, those working in civil society and academia, in Guatemala and Panama.

Two case studies were conducted on the development/adoption of Bill #5504 in Guatemala and Law #570 in Panama.

Data collection and analysis were undertaken in 2020 by a researcher from Guatemala with experience in food policy, with the support of two research assistants from Guatemala and guidance from an international researcher with experience on the CPA of the food industry.

### Publicly available information

#### Selection of our sample of industry actors

Following the first step of the analysis, we used the business database Euromonitor to select the most prominent food industry actors of package products and beverages from Guatemala and Panama, based on their market shares in 2018. We also included in our sample major trade associations in the food industry in both countries, such as the Guatemala Food and Beverages Chamber, the Panama Chamber of Commerce, Industry and Agriculture (CCIAP) and the Central American Food and Beverages Coalition. The latter was included since it comprises food industry actors from both countries.

#### Data collection

We collected information that was published between January 2018 and December 2019, which is the period where both policies were in construction or in discussion, across various sources as recommended by the protocol of the International Netword for Food and Obesity/non-communicable Diseases Research, Monitoring and Action Support (INFORMAS)^(23)^:

- *Food industry material*: national websites and social media accounts (Twitter, Facebook and Instagram) for our sample of industry actors;
- *Government materials*: websites of departments and other agencies responsible for health and education; websites of the Parliament and Senate; register of lobbyists; websites of the political parties involved in public policies; websites of electoral commissions;
- *Materials from universities and professional bodies in health and nutrition:* websites of top five universities in each country with school/departments of nutrition/dietetics; websites of professional associations; websites of major conferences on diet, public health or physical activity;
- Google and Google News.

We created a data collection tracker using an Excel spreadsheet. The spreadsheet had information on date of search, source of information, link to the webpage, name of industry actor, CPA strategy, CPA practice and any other observation. Our data are presented in supplementary material 1.

### Semi-structured interviews

To triangulate the above data and gain deeper and new insight into the CPA strategies of the food industry, we carried out interviews with key stakeholders who interacted directly with food industry actors during the development of Bill #5504 in Guatemala and Law #570 in Panama. Interviews were conducted between April and July of 2020 by two research assistants. We recruited policymakers (former and current), academic experts and representatives from civil society organisations, based on their involvement in technical boards and advisory committees for the development of these policies and their experience in the discussion of both public policies with food industry actors. For recruitment, we send an invitation letter by email to each stakeholder with follow-up by email. Some participants were contacted by the first author and her colleagues at her institution, through their personal networks. Other contacts were obtained from the websites of potential participants or the websites of their organisations. People currently working within the food industry were excluded from the study because of their vested interest in that discussion, with a sense that they will not have a critical opinion of the CPA strategies discussed here^(20)^. We invited eleven individuals in Guatemala and fourteen in Panamá. Eight individuals in Guatemala (two from academia, four from government and two from international organisations) and seven in Panamá (two from academia, four from government and one from international organisation) agreed to participate.

The protocol of the study was approved by the Ethics Institutional Committee of the Institute of Nutrition of Central America and Panama. Upon agreement and after having read a plain language ethical statement about the study, each participant signed an informed consent form prior the interview. The interview guide included semi-structured, open-ended questions previously used for that type of study in similar countries^(26)^ and were adapted for the context of the present study and for the specific public policies in discussion.

Interviews were transcribed semi-*verbatim* by two research assistants. All sensitive information such as participant’s identifiable information was eliminated at that stage. All information collected during the interviews was recorded in an Excel spreadsheet, but not shared here to preserve the anonymity and confidentiality of our participants.

### Data analysis for both methods

For our analysis, we used a conceptual framework of the CPA of the food industry published by Mialon et al. in 2018^(22)^ (adapted from a framework used to classify the CPA of the tobacco industry), in a deductive process. The analysis was simultaneous to data collection in the case of public data and as recommended by INFORMAS, and done after data collection in the case of our interviews.

Before undertaking data collection and analysis, the researchers developed a training operations manual with specific definitions and examples for each CPA strategy to get familiar with the CPA classification. Additionally, the manual provided details on the methodology for searching information on social media, newspaper articles and the websites of the government, universities and professional bodies. Secondly, a pilot exercise was performed by two research assistants with support from the last author, using ten examples of CPA practices from public information data, for internal validity purposes.

The research team held meetings every 2 weeks. Agreement about the classification of examples collected into the different CPA strategies was reached after discussion during those meetings and through email discussions. The first and last author each reviewed a random sample of the results (10 % of all data for publicly available information and 100 % of the data from the interviews) for data quality assurance.

We added new columns in our Excel database where new CPA practices, not identified in the literature, were collected. Tables 1 and 2 therefore present new CPA practices such as one instrumental strategy (development and promotion of private–public initiatives for self-regulation) and two discursive strategies (good governance and contraband).

In the Results section, we report on our findings by country and by CPA strategy, as per Mialon et al.’s classification^(22)^. We present the arguments used by the food industry alongside their instrumental CPA strategies, since those arguments are used in the actions of the industry. Any instrumental or discursive strategies made by interviewees but not backed up by other evidence were not presented here. When referring to a specific example of CPA collected in the public domain, a number and letter are given (i.e. G1) and refer to data presented in supplementary material S1 and S2.

## Results

We identified 166 occurrences of CPA between January 2018 and January 2020 through our INFORMAS analysis for both countries. We identified eighty-six and twenty examples of instrumental strategies and forty-nine and eleven examples of discursive strategies in Guatemala and Panama, respectively, from publicly available information. These categories are not mutually exclusive. CPA practice was mostly applied by the Guatemalan Chamber of Food and Beverages in Guatemala and by the Chamber of Commerce, Industries and Agriculture of Panama (Table 3 and online Supplementary material S1). Guatemala was the country with the highest number of CPA found in the public domain (135 total) compared to Panama (31 total).

**Table 3:** Corporate political activity (CPA) of the food industry in Guatemala and Panama, as identified in the present study (categories are not mutually exclusive)

	Instrumental strategies					Total by actor
	Coalition management	Information management	Direct involvement and influence in policy	Legal strategies	Discursive strategies	
**Food industry actor**						
**Guatemala (Country Total = 135)**						
Interamerican Pepsicola of Guatemala	0	0	2	0	2	4
Coca Cola of Guatemala S.A.	0	0	2	0	3	5
Nestlé S.A.	0	0	0	0	1	1
Bimbo	0	0	1	0	2	3
Guatemalan Chamber of Industry	1	0	0	0	0	1
Guatemalan Chamber of Food and Beverages	7	42	0	0	28	77
Food and beverage trade-association of the Chamber of Industry of Guatemala	2	3	0	0	0	5
ALAIAB-Latin American Alliance of Food and Beverage Industry Associations	0	3	0	0	8	11
Central American Coalition of the Food and Beverage Industry	5	10	8	0	5	28
Total by CPA strategy	15	58	13	0	49	
**Panama (Country Total = 31)**						
La Doña Panamá	0	0	2	0	1	3
National Assembly of Panama	0	1	2	0	0	3
Chamber of Commerce, Industries and Agriculture of Panama	2	2	4	0	4	12
FECAMCO- Federation of the Chambers of Commerce of the Central American Isthmus	0	0	1	0	0	1
H. Tzanetatos Inc	1	1	0	0	0	2
Industrial Labor Union of Panama	0	0	4	0	6	10
Total by CPA strategy	3	4	13	0	11	

The most observed instrumental strategies in both countries were the establishment of direct relationships with policymakers and direct lobbying against the introduction of the proposed public policies. Discursive strategies included criticising ‘lack of evidence’ on both policies, negative impacts on the economy and also pointed at individuals for making their own food choices, in order to shift the focus away from the role of its products in contributing to ill health. Additional examples were shared during our interviews in both countries.

### Guatemala: Bill #5504 on the promotion of healthy eating

Bill #5504 started developing in 2017 and submitted to the Congress by the Parliamentary Front against Hunger in October 2018. As November 2021, the Bill has not yet been discussed in the Congress. Bill 5504 ‘Promotion of Healthy Eating’ proposed to introduce a new nutrition FOPL system, made of warning labels on food and beverages with excessive amounts of sugar, total fat, saturated fat, trans fat and sodium, and marketing regulations targeted to children.

#### Coalition management

We found that even prior to the submission of the Bill, the food industry established relationships with other industry actors, health organisations, communities and the media to discuss labelling norms, regional regulations and new FOPL systems [G14, G21, G23, G78, G79, G106, G110, G11]. In anticipation of such an initiative, the food industry first built alliances within the industry and with other corporate actors to discuss FOPL in the context of the Codex Alimentarius, a non-mandatory compilation of food labelling norms and standards recommended by the Food and Agriculture Organisation (FAO) and the WHO [G14]. For example, on 6 September 2018, the director of the Guatemalan Chamber of Food and Beverages, along with representatives from all the trade associations from the seven Central American countries, participated in a meeting of the Central American Food Coalition to review the progress of the Codex Alimentarius and regional initiatives on FOPL [G14].

In February and March 2019, once the Bill was submitted, the food industry particularly insisted that the government should have used Codex guidelines and standards to develop its new FOPL system, since Codex mandates that the development of standards (such as FOPL) be done by the government in collaboration with other stakeholders, including the food industry[G17]. The Central American Coalition of the Food and Beverage Industry also defended the same argument [G103].

After the Bill was submitted, in January 2020, these industry actors continued to have internal discussions on FOPL, with the Guatemalan Chamber of Industry participating in an International Regulatory Perspectives Forum where the topic of FOPL, in Latin America, and its impacts on the reputation of the products sold by the industry were discussed [G11]. The Guatemalan Chamber of Food and Beverages also had a ‘Labeling Committee’, whose purpose was ‘to analyse the national and international law initiatives, government agreements and regulations that involve the labelling of Food and Beverages’ [G12]. Similarly, the Central American Coalition of the Food and Beverages Industry had labelling technical committees whose aim was to develop country positions on the topic for a ‘fair and balanced’ approach, in order to avoid obstacles to the development of the industry in the region [G91].

Our interviewed participants noted that the media in Guatemala, which is close to the food industry, may have helped in promoting the industry’s position against the Bill #5504

‘I saw a couple of media sources, one of them, Emisoras Unidas [a radio station], had a broadcaster saying that he did not agree with the Bill. I think that it was misinformation, but a guided misinformation. […] I know that [in the Bill discussions] the food industry developed a list of the [negative] consequences for each article of the Bill. Even though the public health advocates clarified all that unfounded information, the industry disseminated it’ [Government worker]

One of our interviewed participants noted that the personnel from the Embassy of the United States of America, also opposed the Bill, arguing that if the initiative was approved, there will be losses in investments for the country, although we cannot confirm whether that position from the Embassy was influenced by the food industry:

‘There was an invitation to attend a lunch at the home of the Ambassador of the United States and (…) the Commercial Attaché [talked about] the initiative, how bad the initiative was and that the United States was not going to support [it]. [He asked] how possible it was that this type of initiative was being proposed in this country (…). I remember [he said that] the industry is no longer going to invest here in Guatemala’ [Staff from an international organisation]

Our interviewed participants also described how the food industry tried to discredit the position of public health advocates during the discussions in the Congress.

Beyond the specific influence on the FOPL discussion, our interviewees also explained that the food industry was proactive in nurturing relationships with third parties; relationships that could then be used to protect or promote the industry’s interests. For example, one interview participant [academia] also indicated that government officials are sometimes appointed by the food industry to work in health institutions, which may have helped in having the industry’s position represented in those institutions. The food industry also provides scholarships for the education of student and undertakes community initiatives, including those on physical activity – thus building a good image for itself in communities.

‘There are prizes and scholarships for primary, secondary and high schools and university degrees. […] The Castillo Foundation, that is linked to the entire beverage industry, is sponsoring education’. [Academic]

‘They have always supported sport related activities, but at that time [during the FOPL discussion], if you check the newspapers, maybe you can see that there was more [on the] support to those activities’. [Academic]

#### Information management

We identified CPA examples where the food industry amplified information that questioned the Bill and tried to suppress information that supported the Bill and the scientific evidence from which it is based. During discussions of the Commission on Social Development in the Congress, the Guatemalan Chamber of Food and Beverages promoted its preferred FOPL system, the Guideline Daily Amount (GDA) [G18, G112, G113] and discredited the proposed Bill [G15, G20, G24, G25, G26, G36, G37, G39, G41, G42, G46, G52, G54, G55, G61, G62, G63, G64, G66, G68, G72, G73, G74, G76, G80, G96, G97, G98, G99, G100, G102, G104].

When the FOPL Bill was submitted, the Guatemalan Chamber of Food and Beverages questioned its content, which may have spread misinformation [G35, G51 G53], but clarifications were soon provided by different organisations that supported the initiative. The Guatemalan Chamber of Food and Beverages, for example, indicated that behind the ‘good intention’ of the Bill for having a healthy lifestyle, ‘in practice what it does is that it restricts our freedom, imposing on us the vision of enlightened technocrats who despise freedom’ [G51]. The industry misleadingly claimed that the Bill would increase food costs, through a tax on foods and beverages [G35], which in reality there is no such tax included in the Bill [G53].

The main arguments used by the food industry to support its position were: (1) the warnings labels were not aligned with international norms, like those of the Codex Alimentarius and those of the Central American Technical Regulation (RTCA), in which the Central American Ministries of Economy proposed the use of GDA; (2) the image of the industry’s products would be unnecessarily damaged; (3) there was no scientific evidence on the role of ultra-processed foods as contributing to ill health; hence, the Bill would not be effective at reducing the levels of obesity and NCD; (4) the main public health problem in Guatemala is undernutrition, not obesity; (5) the Bill would promote smuggling [G25, G54, G65, G74]; (6) FOPL would confuse consumers; (7) the Bill promotes the consumption of traditional foods, some of which increase the risk of developing obesity and NCD; (8) the Bill would increase the cost of food products; (9) the Bill would have serious negative effects on the economy and employment.

More specifically, the industry for example claimed that the Bill, if introduced, would increase the cost of products most consumed by the poor, hence, lowering their standards of living [G53]. Food industry actors also claimed that reformulating products so that they would not have warnings would increase the cost of such products, this having a negative impact on the economy, local manufacturers and importations, which would lead to unemployment, a decrease of economic activity and even less taxes revenues for the government [G16, G32, G33, G34, G53, G60, G65, G109]. The food industry argued that the use of warning labels is not based on evidence; however, we could not find any scientific peer-review references cited by food industry actors to support any claim. Food industry actors therefore claimed that there was a lack of evidence to support the proposed warning labels, but did not provide any scientific evidence to defend this argument.

Moreover, our participants noted that during Bill discussions, the food industry tried to frame the debate, shifting the blame away from the role of its products in contributing to ill health and instead focusing on the importance of individual responsibility and healthy lifestyles.

‘[The food industry] argued that ultra-processed products were not the ones that were causing the prevalence of non-communicable chronic diseases [it was due to the] lack of physical activity. [Also that] education had to be strengthened […]’. [Government official]

That argument about individuals being responsible for their own choices was also used by the industry to argue that it was already doing something: PepsiCo, Coca-Cola, Nestlé, Bimbo and national and regional Chambers of Foods and Beverages claimed that existing labels already provide clear information to individuals [G2, G4, G6, G8, G9, G10, G17, G28, G43, G57, G58, G97, G104]. For example, Pepsi Cola declared: ‘We want our consumers to make informed decisions for themselves and their families, for that reason we include clear information about the ingredients of our products on the labels’ [G2]. Many of these arguments were unfounded or distorted existing evidence. We provide counterarguments to these claims in our discussion section.

The food industry also focussed on processed foods (most of which the Bill did not target) instead of ultra-processed ones (which consumption is problematic). The industry cherry-picked data to support its claims, arguing for example that only a ‘small’ proportion of ultra-processed products are consumed by the population, relative to the total amount of foods consumed [G74].

‘[The food industry claimed that] of everything that the population eats, only 20 % comes from processed foods, [so then] why this initiative? (…) So, it was real information, but they were using it to their advantage […]’ [Academic].

To further promote its position, in May 2018, the Guatemalan Chamber of Food and Beverages launched the campaign ‘Vida Saludable’ (‘Healthy life’). The campaign took place in the ‘midst of several initiatives promoted in the Congress of the Republic that seek to increase controls on [processed foods], supposedly be responsible for diseases such as diabetes and cancer, among others’ [G27, G29, G46]. The director of the Chamber explained that the campaign aimed to provide objective information to the population about processed foods: ‘there is bad information about processed products saying that they cause cancer’ [G28]. The Chamber also published an online Bulletin on ‘myths’ about ultra-processed products, which were to be targeted by the Bill (most ultra-proccessed food products would have had to carry a FOPL if the Bill was introduced), claiming for example that the consumption of those products is not associated with an increased risk of developing NCD (that claim is contrary to best available evidence)^(7,8)^ [G13, G44].

A scientific forum was organised by the Association of Chemists and Pharmacists of Guatemala after the Bill was submitted. The aim of the forum was to discuss the scientific background for the Bill 5504. In that forum, actors from the food industry were present and promoted their position vocally and intimidated individuals defending the Bill.

‘The food industry intimidated people who were defending the initiative. (…) there was a lot of opposition from people who work in the industry (…) I think (…) it was [a kind of] intimidation (…). I saw that they used that a lot. Especially in a country like ours, where there is so much insecurity. [The intimidation] was verbal, in those kinds of meetings, like in this forum – [the forum] was very good and [the initiative was] defended. I think it was one of the best forums at that time’. [Academic]

‘There [in the forum] it seemed to me that there was a bit of intimidation, having sent such a large group of them [food industry representatives] to fill the back of the auditorium and put some pressure and as observers of the process’. [Academic]

#### Direct involvement and influence in policy

We found evidence that the food industry directly lobbied Members of the Congress and the Vice President of the Republic against the Bill, as reported by a government official:

‘They [the food industry] reached the Presidency, there was some communication from the Vice President to investigate [their requests] ….[he wanted] to find out how the situation was, […] what was happening’. [Government official]

We also found evidence of the use of the ‘revolving door’, when a former food industry employee goes to work for the government or vice versa. Our interviewees noted that industry-friendly officials worked in the Department of Food Control and Regulation of the Ministry of Health:

‘The director of the Department of Food Control and Regulation (…) came from the food industry’. [Academic]

It is important to note that the opposition to the Bill also came from within the government.

‘[Businesses are] definitely in charge of [appointing] the officials of the Ministry of Economy and the Ministry of Finance, but they also have interference in other Ministries such as the Ministry of Agriculture, and also on certain aspects in the Ministry of Health. If [businesses] do not agree with the measures taken by the Ministry of Health, they try to replace [the officials there with] people who are more aligned to their ideology’. [Government official]

This may have led to internal influence, for example, over the decisions taken by the technical board working on the development of the FOPL in the same Ministry.

‘An officer from the department of Food Control and Regulation of the Ministry of Health sent a lawyer and a nutritionist from that unit, to support the whole team [who was working on the Bill in the Ministry of Health]. When they finished [drafting] the Bill, he said he was sorry, but he did not agree with everything that had been worked on.’ [Staff in an international health organisation]

‘At some point [the industry] sought to [participate in the initiative]. And the person who asked for it was this person (…) from the Department of Food Control and Regulation. He wanted a table that included the food industry.’ [Academic]

The industry used those internal differences to argue that the Ministry of Health (through the Department of Food Control and Regulation) did not support the Bill.

‘A lawyer [from the food industry] showed a letter signed by Ministry’s personnel [from the department of Food Control and Regulation]. [In the letter,] the Department of Food and Regulation [indeed said they] supported the industry against the initiative.’ [Government official]

Moreover, the former Ministry of Economy was the Executive Director of the Guatemalan Chamber of Food and Beverages (in 2020) [G14, G24, G28, G30, G32], which might have opened doors for the industry in that Ministry.

‘I think we all know it and it is not a secret that a former Ministry of the Executive [Minister of Economy] now has become the Director of the Guatemalan Chamber of Food and Beverages’. [Government official]

Some food industry companies, such as PepsiCo, Coca Cola and the Bimbo Group, in their lobbying against the Bill, claimed that they already complied with local food labelling regulation thus implying that there was no need for the Bill [G1, G2, G3, G4, G5, G6, G7, G9].

Other industry actors supported voluntary measures instead of mandatory regulation on FOPL, with, for example, the Central American Coalition of the Food and Beverage Industry referring to the Food Labeling Central American Technical Regulation:

‘The government of Costa Rica (…) proposes an evolution in the current regulation, towards a front-of-pack nutrition labeling system on foods and beverages, which [uses] the Guidelines of Daily Amount (GDA) – which today is widely used on a voluntary basis by much of the formal industry in the region’. [G105]

### Panama: Law #570 on the taxation of sugar-sweetened beverages

In Panama, Law #570 aimed at implementing a SSB tax of 7 % for sodas, 10 % for syrups and concentrates for SSB production and 5 % for the rest of SSB. The Law was presented in the Congress in September 2017 and approved in February 2019. Supplementary Material S2 provides details for each CPA strategies identified from public available information.

#### Coalition management

In Panama again, the food industry built alliances with third parties to support its position against the increase in the taxation. Within the industry, corporations acted through the Chamber of Commerce, Industries and Agriculture of Panama. Indeed, 2 days before the Law was approved, the Chamber organised a meeting with its internal working group in order to discuss law #570 [P4].

#### Information management

As in Guatemala, the food industry in Panama used science to its advantage. During the discussion period for the development of the Law in Congress, the food industry indeed presented negative results on the impacts of the proposed SSB taxation, with data that came from industry-funded economists.

‘[The food industry] presented [studies of SSB taxation funded by them]. [During the discussions] there were two or three […] economists and they said that they were financed with funds from the Chamber of Commerce to make projections of the negative effects of the initiative. They were directly linked to the Chamber of Commerce.’ [Government official]

During the same discussion period in congress, the food industry also used industry-funded research articles that are not free of conflicts of interest.

‘[There were] some [scientific] articles (…) [but the] funding came from Coca-Cola.’ [Government official]

Our interview participants indicated that the food industry further highlighted the negative effects the taxation would have using Mexican data, although the evidence presented in reality supports the adoption of an SSB tax.

‘When the law initiative went to the second debate [in Congress], (…) an INSP [National Institute of Public Health in Mexico] report had come out about what was happening in Mexico [regarding the SSB tax]. […]. The report said the tax has no significant reduction on employment. I clearly remember that [the food industry] took two lines of this report saying that the initiative would have a negative effect on employment. (…) they took some parts of those reports (…) and changed and distorted [the information].’ [Government official]

The food industry argued that if the Law was introduced, it would lead to the loss of 24 000 direct and indirect jobs [P17, P19]. One of our interview participants mentioned the strong influence of the food industry on the government when using this argument:

‘The food industry has very strong, long and powerful tentacles, so the industry put pressure on national authorities, especially with the threat of job losses. We learned that the threat of job losses is one of the most vulnerable points for the people who are working in the government. So, the authorities had the tendency to give in.’ [Government officials]

The President of the National Council of Private Enterprise (CoNEP) and a representative of the Chamber of Commerce, Industries and Agriculture of Panama used other arguments against the proposed tax increase: (1) the increase would negatively impact the economy as it will raise the price of SSB for the consumer and (2) the tax will not resolve the obesity problem [P12].

Moreover, food industry actors also criticised independent results presented by the Ministry of Health during the development of the Law, arguing that the Ministry did not have the relevant expertise.

‘They said that [there were no technicians] in the field of economics. (…) They constantly discredited the information [that the Ministry of Health presented].’ [Government official]

Finally, and similarly to the arguments used in Guatemala, the Industrial Labor Union of Panama and the Chamber of Commerce, Industries and Agriculture of Panama minimised the role that the consumption of SSB has on obesity and diabetes and promoting alternatives to the regulation of beverages: more education, increased physical activity and balanced diets (that could include SSB) [P10, P12, P16, P18, P21, P23, P24].

‘[The food industry argued that the Ministry of Health was not] doing the job it [had] to do, which was educating the population - which was getting children to do physical activity.’ [Government official]

‘[The food industry] said that they could fund a nutrition education program. With this everyone would be aware of how important it is to eat healthy foods. They said that we did not need the Law initiative, that without the initiative they were willing to collaborate.’ [Academic]

The food industry used its own platforms to disseminate that information. For example, in February 2018, during the development of the Law, the food industry launched a forum called ‘Juntos por la Nutrición’ (‘Together for Nutrition’) [P11, P14]. The Chamber of Commerce, Industries and Agriculture of Panama (CCIAP) and the Industrial Labor Union of Panama (SIP) organised the forum to discuss the impacts their products have on health and to discuss examples of public–private initiatives that they claimed benefit health, as well as intersectoral work opportunities for promoting population well-being [P14]. The Ministry of Health and other organisations attended the forum [P11].

#### Direct involvement and influence in policy

In addition to trying to influence the debate by using information on SSB taxation, the food industry has been proactively influencing the policy space in Panama.

During discussions in the Congress, food industry actors for example threatened to withdraw their investment from the country. Moreover, the food industry made sure that its employees were present during the discussions in Congress and confronted policymakers, arguing that if the Law was adopted, their jobs would be lost.

‘One actor from the food industry said that 5000 [jobs] were going to be lost. So they wanted to confront us with their workers, with the trade union.’ [Government official]

‘When the Law initiative was presented, the industry took its workers to the Court so they could say that if the initiative was adopted, they would be out of work. There were workers who lent themselves to [speak] in favor of the industry.’ [Government offcial]

The Industrial Labor Union of Panama and the Chamber of Commerce, Industries and Agriculture of Panama moreover sought to be part of the government’s commission which verifies the final destination of the revenues raised from the SSB tax [P22]. In article No. 5 of the approved law, in fact, established the creation of the ‘Commission for the Improvement of Health’ which includes representatives from both industry organisations aforementioned^(16)^.

We identified examples of food industry’s direct lobbying the President of the Republic, before and after the law was approved, which had delayed its implementation [P3, P6, P7, P8, P15, P26]. The food industry had its position shared in opinion pieces in the media [P8, P26] and sent public letters to the President seeking to veto the Law [P6].

‘The industry told the President that they wanted to participate in the technical boards, in working groups or technical groups to contribute to the initiative. They sought participation directly at the head.’ [Government official]

At that time, the President of Panama (until July 2019) was also a businessman from the alcohol industry. In February of 2019, the President vetoed the Law #570 [P3, P15].

‘At the time, the President of the Republic was a businessman, a beverages entrepreneur, alcoholic beverages in that case. We knew that there was direct communication between some businessmen of the same related business or field. They were in direct communication with the President (…) maybe they had influence in many things.’ [Government official]

‘The President at that time came from the business sector. He had important links with the food industry. On many occasions, [the food industry said to the Ministry of Health]: I am going to talk with (…) the President of Panama. They told us that they had direct contact with him.’ [Government official]

Our interviewed participants noted that the influence of the food industry over the development of the SSB tax resulted in major changes to the final content of the Law, with a reduction in the originally proposed tax rate from 20 % to the approved rate of 7 %.

## Discussion

For our two case studies on the introduction of a new Bill on the Promotion of Healthy Eating in Guatemala, and the adoption of SSB taxation Law in Panama, we found evidence of the CPA strategies used by the food industry to interfere with the development of these public health policies in both countries, especially from publicly available information. Our results suggest that, independently of the public policies under discussion, food industry actors in the region use similar tactics and practices.

### Guatemala

We found that the food industry first built strategic alliance with third parties in Guatemala. We also observed that the Guatemalan Chamber of Food and Beverages was the food industry actor with the most CPA in the country. This actor represents the food industry which might explain more access to resources to perform a variety of actions. Specially, they use the ‘information management’ strategy. Here, the industry questioned the evidence on which the government based its development of Bill #5504^(27–29)^. Contrary to the arguments used by the industry, as described in our results section, Guatemala has the highest prevalence of the double burden of malnutrition in the Western hemisphere^(30)^ and overweight and obesity prevalence in women of reproductive age exceeds the prevalence of stunting in children under 5 years^(31)^.

There is growing evidence supporting the introduction of warning labels^(32–36)^. Epidemiological evidence has demonstrated that front-of-pack warning labelling (FOPWL) systems, which allow consumers to quickly, easily and correctly identify products that are excessive in critical nutrients, are an effective health policy tool to improve consumer’s understanding, perception and purchase decisions, helping to tackle unhealthy diets and promote healthier food environments^(32–36)^. Chile, Peru, Uruguay and Mexico have implemented octagonal warning labels. In Chile, FOPWL system has proven to reduce purchases of SSB and breakfast cereals^(36)^ and have been projected to prevent 1·3 million cases of obesity in Mexico over 5 years^(34)^.

On the other hand, contrary to food industry arguments, neither RTCA nor Codex impedes the implementation of non-discriminatory measures to protect public health and ensure population’s health^(37)^. Despite all the striking evidence about the effectiveness of FOPWL in the Latin American region, studies in Colombia, Brazil and Chile found that the food industry used similar arguments in those countries, which suggests the companies might align their responses and arguments across the region^(38–42)^. The Chilean and Mexican experience highlight the importance of the involvement of civil society as part of the policy process to support accountability and transparency. Civil Society organisations can help counter the influence of the food industry^(43)^. The presence of civil society during the political process, however, has not been particularly important in Guatemala, which might explain, to some extent, the slow adoption of food policies to prevent obesity and NCD^(44)^.

These results for Guatemala are consistent with similar studies on the food industry interference in the Latin American region. Recent evidence about the introduction of warning labels in Mexico, Chile, Peru and Uruguay showed that the food industry had a privileged access to government officials which led to the delay and modification in the proposed regulations, making them more favourable to the industry interests^(45)^. Another study commissioned by Pan American Health Organization, and that included Guatemala, found that food industry actors tried to influence policymakers when public health policies were discussed^(21)^.

### Panama

An important finding in Panama was the evidence of lobbying at the highest level of governance, with the industry having direct contacts with the President of the Republic, resulting in a temporary veto of the SSB tax, after its approval in 2019. This success at influencing the policy process for the industry might explain the lower number of CPA strategies found in Panama compared to Guatemala. Our findings in Panama are aligned with similar cases in the region (Chile and Peru) where pro-business governments have stronger ties with the industry and where the implementation of public health policies is delayed^(45)^. Similar to what we found in Guatemala, the food industry claimed that there was a need for people to do more physical activity to decrease the levels of obesity; however, there is overwhelming evidence on the causal relationship between SSB consumption and obesity, metabolic syndrome and diabetes^(28,29,46)^. The food industry actors also claimed that the proposed increase in the SSB tax would have a negative economic impact for the country, especially due to reductions in employment. However, to date, there is no evidence to support that claim in countries that have adopted such policies^(47)^. Moreover, there is evidence that SSB taxation helps reduce diabetes and cardiovascular events incidence in simulation studies^(48)^, thus saving money for the public health system in the long term^(49)^. In its use of economic arguments, the food industry used studies and authors funded by the SSB sector and cherry-picked data that favoured its interests. With a tax at 7 % (instead of the initial 20 % that was proposed in the Bill), the industry absorbed the differential cost in order to maintain the same retail price; hence, the tax might not affect purchase behaviour.

### Strengths and limitations of the study

The key limitation of our study was the low response rate (50 % in Panama and 72 % in Guatemala) and therefore our small number of interviewed participants. As a result, there are potentially more examples and practices of CPA performed by the food and beverage industry in both countries that we could not document. This is particularly important in the case of Guatemala, since as in May 2022 the Bill has not been discussed in the Congress. To some extent, we believe that the global health crisis of Covid-19 during 2020 affected participation, with pressure on public health experts to work on the health crisis in their countries, especially at the time of data collection. The main strength of our study is the combination of two qualitative methods that allowed us to triangulate the information found. Another added value to the literature is that this is the first study, to our knowledge, investigating the CPA practices of the food industry in Central America with a particular focus on two public health policies.

Both Guatemala and Panama are facing major challenges related to the increasing prevalence of NCD^(50)^ and stronger public policies are clearly needed to protect population health. There is an urgent need to strengthen local capacity and support governments to respond to ongoing and future undue influences on the food industry. We anticipate a momentum where other public health policies and programmes will be developed and implemented in the short term in Central America (e.g. the Central American FOPL technical regulation). The results of our study might serve to identify the arguments and actions that are put forward by the food industry in its opposition to such public policies. This might help in preparing counterarguments as well as fostering policy dialogue and relationships with policymakers and civil society organisations.

## Supplementary material

For supplementary material accompanying this paper visit https://doi.org/10.1017/S1368980022001811. click here to view supplementary material
