# Supplementary material for: Two countries, similar practices: the political practices of the food industry influencing the adoption of key public health nutrition policies in Guatemala and Panama
Source: Public Health Nutr. 2022 Aug 22;25(11):3252–64. doi: 10.1017/S1368980022001811 (PMC9991768; doi:10.1017/S1368980022001811)
Supplement: Supplementary file 1 [file S1368980022001811sup.zip › S1368980022001811sup002.docx]

Supplementary material S2. Corporate Political Activity from publicly available data by food industry actor in Panama (2017-2019)

| **Code** | **Food industry actor** | **Source** | **Strategy** | **Code** | **Data coded** | **Notes** | **Website URL** | **Date collected** |
| --- | --- | --- | --- | --- | --- | --- | --- | --- |
| P1 | National Assembly of Panama | Website | Information managenent | Supression | Por considerar el impacto positivo a la salud, diputados cerraron fila para aprobar por insistencia, en segundo debate, el proyecto de Ley 570 que promueve un estilo de vida preventivo a través de un bajo contenido de azúcar en las bebidas endulzantes. La citada normativa, que fue objetada por el Ejecutivo, crea el plan de acción para mejorar la salud y se dictan otras disposiciones para establecer el impuesto selectivo al consumo de bebidas azucaradas y los criterios para su uso. Una de las objeciones se basó en que el Proyecto 570 violaba reglas del comercio internacional y la seguridad jurídica al establecer que el etiquetado del producto debía estar en español. Al respecto, el pleno decidió que una vez se promulgue la ley, se dará un plazo de 90 días a las industrias para adecuar el tema del idioma español en el etiquetado de los productos que entran a Panamá. |  | https://asamblea.gob.pa/noticias/pasa-tercer-debate-proyecto-de-bebidas-azucaradas | 04-ago-20 |
| P2 | National Assembly of Panama | Website | Direct involvement and influence in policy | Direct actors | La presidenta de la Asamblea Nacional, Yanibel Ábrego, una de las proponentes, en conjunto con el colega Javier Ortega, señaló que el Órgano Legislativo esta aprobando un proyecto “noble” [Ley 570] que vela por las mejores condiciones de vida de los panameños y que el documento resultó de acuerdos con todos los actores empresariales y del sector salud. |  | https://asamblea.gob.pa/noticias/pasa-tercer-debate-proyecto-de-bebidas-azucaradas | 04-ago-20 |
| P3 | National Assembly of Panama | Website | Direct involvement and influence in policy | Indirect access-lobbying | [La presidenta de la Asamblea Nacional, Yanibel Ábrego] dijo que por muchos meses se trabajó arduamente en la propuesta [Ley 570 ] y manifestó tener tristeza al vetarse, sobre todo por el tema del etiquetado. |  | https://asamblea.gob.pa/noticias/pasa-tercer-debate-proyecto-de-bebidas-azucaradas | 04-ago-20 |
| P4 | CCIAP-Chamber of Commerce, Industries and Agriculture of Panama | Facebook | Coalition management | Intern | Cámara de Comercio, Industrias y Agricultura de Panamá 19 de febrero de 2019 · Público El Grupo No.2 de Alimentación de la CCIAP, realizó una reunión, con la finalidad de abordar la Ley No. 570 que establece el impuesto selectivo a las bebidas azucaradas y otras disposiciones | La información se encontró nuevamente en el Instagram de la Cámara de Comercio, Industrias y Agricultura de Panamá https://www.instagram.com/p/BuFIa2WHRDo/ https://www.instagram.com/p/ByVbQG-hzV9/ | <https://www.facebook.com/photo/?fbid=2859867880690339&set=a.287901447887008> | 21-may-20 |
| P5 | CCIAP-Chamber of Commerce, Industries and Agriculture of Panama | Facebook | Direct involvement and influence in policy | Indirect access-lobbying | Cámara de Comercio, Industrias y Agricultura de Panamá 22 de mayo de 2019 · Público Miembros del Grupo 2 de Alimentación sostienen conversatorio con la Dra. Anarella Jaen (Anarella Jaén de Merón), jefa nacional del Departamento de Protección de Alimentos del MINSA Panama y su equipo. |  | <https://www.facebook.com/photo/?fbid=3071611069516018&set=pcb.3071612582849200> | 21-may-20 |
| P6 | CCIAP-Chamber of Commerce, Industries and Agriculture of Panama | La Prensa, Panamá | Direct involvement and influence in policy | Indirect access-lobbying | La Cámara de Comercio, Industrias y Agricultura de Panamá (Cciap) pidió al presidente de la República, Juan Carlos Varela, el veto parcial al proyecto de ley No. 570 "que crea el plan de acción para mejorar la salud y dicta otras disposiciones para establecer el impuesto selectivo al consumo de bebidas azucaradas y los criterios para su uso". La petición la hizo ayer, 21 de febrero, a través de una misiva. | La información se encontró nuevamente en el periódico "El Economista" https://www.eleconomista.net/actualidad/Empresarios-piden-a-presidente-panameno-vetar-ley-que-tasa-bebidas-azucaradas-20190222-0009.html | <https://www.prensa.com/politica/CCIAP-parcial-proyecto-impuesto-azucaradas_0_5243475607.html> | 28-jul-20 |
| P7 | CCIAP-Chamber of Commerce, Industries and Agriculture of Panama | La Prensa, Panamá | Direct involvement and influence in policy  Discursive strategy | Direct actors Frame the debate | La Cámara de Comercio, Industrias y Agricultura de Panamá (Cciap) pidió al presidente de la República, Juan Carlos Varela, el veto parcial al proyecto de ley No. 570[...] En la carta, la Cciap reiteró su rechazo al nuevo impuesto. La organización insistió en que durante la discusión del proyecto de ley planteó que se debía educar a la población panameña en la promoción de planes de acción que incentiven hábitos de vida saludable. Para lograrlo, planteó el establecimiento de un fondo público-privado con el fin de lograr estos objetivos. | La información se encontró nuevamente en el periódico "El Economista" https://www.eleconomista.net/actualidad/Empresarios-piden-a-presidente-panameno-vetar-ley-que-tasa-bebidas-azucaradas-20190222-0009.html | <https://www.prensa.com/politica/CCIAP-parcial-proyecto-impuesto-azucaradas_0_5243475607.html> | 28-jul-20 |
| P8 | CCIAP-Chamber of Commerce, Industries and Agriculture of Panama | La Prensa, Panamá | Direct involvement and influence in policy | Indirect access-lobbying | La Cciap indica que "es de suma preocupación lo descrito en el artículo 13 del proyecto de ley No. 570, que en su tenor señala que, a partir de la entrada en vigencia de esta ley, la industria de bebidas azucaradas deberá incluir en todos sus productos nacionales e importados el contenido nutricional en su etiquetado, y deberá estar en idioma español”, por lo cual le solicita a Varela que vete el artículo y que el Ministerio de Comercio e Industrias sea quien establezca los parámetros del etiquetado. La solicitud de veto al artículo 13 se fundamenta en la nota enviada por el Ministerio de Comercio e Industrias al pleno de la Asamblea Nacional, la cual guarda referencia al etiquetado, señalando que dentro del marco del proceso de incorporación de la República de Panamá al Subsistema de Integración Económica Centroamericana (SIECA), este tema parte de las negociaciones en curso. | La información se encontró nuevamente en el periódico "El Economista" https://www.eleconomista.net/actualidad/Empresarios-piden-a-presidente-panameno-vetar-ley-que-tasa-bebidas-azucaradas-20190222-0009.html | <https://www.prensa.com/politica/CCIAP-parcial-proyecto-impuesto-azucaradas_0_5243475607.html> | 28-jul-20 |
| P9 | CCIAP-Chamber of Commerce, Industries and Agriculture of Panama | La Prensa, Panamá | Discursive strategy | Costs | Tras la aprobación de este proyecto[Ley No. 570], el Sindicato de Industriales de Panamá informó que se opone a los proyectos de ley que creen o incrementen los impuestos a las empresas y consumidores, ya que coloca a la industria en desventaja competitiva con otros países de la región. | La información se encontró nuevamente en el periódico "El Economista" https://www.eleconomista.net/actualidad/Empresarios-piden-a-presidente-panameno-vetar-ley-que-tasa-bebidas-azucaradas-20190222-0009.html | <https://www.prensa.com/politica/CCIAP-parcial-proyecto-impuesto-azucaradas_0_5243475607.html> | 28-jul-20 |
| P10 | CCIAP-Chamber of Commerce, Industries and Agriculture of Panama | Panamá América | Discursive strategy | Frame the debate | Alimentos procesados, aditivos y desnutrición, en I Foro de nutrición (...)El Foro concluyó con un panel sobre las oportunidades del trabajo multidisciplinario e intersectorial para beneficio de la salud y nutrición de la población. En cuanto a aportes específicos de cara a la obtención de este objetivo el presidente del Sindicato de Industriales de Panamá, Michael Morales, explicó que en el sector industrial se han efectuado ajustes en lo que tiene que ver con el procesamiento, fortificación y etiquetado de alimentos. Igualmente ha habido acciones como campañas para desarrollar en la población encaminadas a que se convierta en propiciadora de su propia salud practicando hábitos saludables de nutrición. |  | <https://www.panamaamerica.com.pa/life-style/alimentos-procesados-aditivos-y-desnutricion-en-i-foro-de-nutricion-1097106> | 29-jul-20 |
| P11 | CCIAP-Chamber of Commerce, Industries and Agriculture of Panama | Panamá América | Information management Coalition management | Amplification Government bodies and other organizations -External | Alimentos procesados, aditivos y desnutrición, en I Foro de nutrición También participaron en el I Foro Juntos por la nutrición la Dra. Itza Barahona de Mosca, directora general de salud del Ministerio de salud; Dr. Fernando Cardini, los consultores internacionales Dr. Fernando Cardini y Dra. Susana Socolovsky, así como el Prof. Rafael Cornes (consultor de Fepale), Ing. Héctor Cori , Msc. Adiana Blanco, Ing. Omaris Vergara, Ing. Frank Tedman, entre otros. Este foro fue organizado por el Sindicato de Industriales de Panamá (SIP) y la Cámara de Comercio, Industrias y Agricultura de Panamá. |  | <https://www.panamaamerica.com.pa/life-style/alimentos-procesados-aditivos-y-desnutricion-en-i-foro-de-nutricion-1097106> | 29-jul-20 |
| P12 | CCIAP-Chamber of Commerce, Industries and Agriculture of Panama | El Economista | Information management Discursive strategy | Suppression Frame the debate | Impuesto a bebidas azucaradas pasa a tercer debate con críticas (...)Severo Sousa, presidente del Consejo Nacional de la Empresa Privada (CoNEP), considera que “la economía local no está en el mejor momento para impuestos nuevos, que lo que hacen es subir el precio al consumidor que igualmente ya está impactado con lo que cuesta hacer su supermercado o en gastos de entretenimiento si los tiene; ya que las bebidas azucaradas incluyen gaseosas, concentrados, jugos, refrescos, etc”. Por su parte, el empresario Frank Tecman, representante de la Cámara de Comercio, Industrias y Agricultura de Panamá, en el debate del proyecto ley en la Asamblea, sostuvo que “un impuesto no va a lograr controlar el problema de la obesidad, porque una persona es obesa inclusive por ansiedad o porque tiene mucho trabajo o porque tiene preocupaciones y come más de lo que quema”. |  | <https://www.metrolibre.com/economia/124675-impuesto-bebidas-azucaradas-pasa-tercer-debate-con-criticas.html> | 29-jul-20 |
| P13 | FECAMCO- Federation of the Chambers of Commerce of the Central American Isthmus | Panamá América | Direct involvement and influence in policy | Direct actors | Expresidente de la Cámara de Comercio Raúl Delvalle asume presidencia de Fecamco. (...)Fecamco representará al Comité Consultivo de la Integración Económica (CCEI), finalizará la propuesta de RTCA nutricional y dar seguimiento a Etiquetado Frontal de Alimentos, seguimiento a la Notificación Sanitaria Obligatoria / Revisiones al RTCA de registros de cosméticos, así como a la Política Regional de Movilidad y Logística y apoyar la promoción de la Plataforma de Incidencias al Comercio, donde a través de este mecanismo se busca un mejor seguimiento y solución a los problemas que enfrentan los empresarios centroamericanos, entre otros. | Noticia relacionada con el Reglamento Técnico Centro Americano (RTCA) y Etiquetado Frontal de Alimentos | <https://www.panamaamerica.com.pa/economia/expresidente-de-la-camara-de-comercio-raul-delvalle-asume-presidencia-de-fecamco-1154957> | 28-jul-20 |
| P14 | H. Tzanetatos Inc | Facebook | Information management Coalition management | Amplification Intern | H. Tzanetatos 14 de febrero de 2018 · Público La Cámara de Comercio, Industrias y Agricultura de Panamá (CCIAP) y el Sindicato de Industriales de Panamá (SIP), organizaron este miércoles, el primer Foro "Juntos por la Nutrición", con la finalidad de analizar, desde el punto de vista técnico-científico, el rol de los alimentos previamente envasados en la salud y nutrición, entre otros temas. Los ejes temáticos analizados en este foro estuvieron relacionados al sector salud y nutrición, situación actual de nutrición en Panamá, papel de los alimentos pre envasados; y ejemplos de medidas público-privadas en beneficio de la salud; oportunidades de trabajo intersectorial para el bienestar de la población. H. Tzanetatos fue parte de los invitados a este interesante foro, el cual tiene gran importancia para nuestro país. #panama #saludpanama #htzanetatos #forojuntosporlanutricion Ver menos |  | <https://www.facebook.com/HTzanetatos/videos/2002580519982474> | 27-may-20 |
| P15 | La Doña | Panamá América | Direct involvement and influence in policy | Indirect access-lobbying | La Comisión de Economía y Finanzas de la Asamblea Nacional aprobó, por insistencia, el proyecto de ley que crea el impuesto selectivo al consumo de bebidas azucaradas. La decisión de los diputados se tomó luego del veto presidencial que devolvió a la Asamblea Nacional dicha propuesta. |  | <https://www.panamaamerica.com.pa/economia/aprueban-por-insistencia-proyecto-sobre-impuesto-bebidas-azucaradas-1131570> | 03-ago-20 |
| P16 | La Doña | Panamá América | Discursive strategy | Frame the debate | Luego que se retomara la discusión del proyecto de ley 570, que establece un impuesto selectivo al consumo de bebidas azucaradas, ha generado reacciones entre los consumidores y economistas, quienes coinciden en que la normativa es positiva y pone un alto al problema que está afectando a la población panameña. La iniciativa busca crear un nuevo impuesto selectivo al consumo de bebidas azucaradas y energéticas del 8% sobre su precio, ya sean importadas o de producción nacional. La obligación de este impuesto se paga al momento de la compra de la bebida azucarada, ya sea en supermercados, abarroterías o distribuidores en general. No obstante, el Sindicato de Industriales de Panamá (SIP) asegura que el tema tiene una óptica diferente, pues se trata de un problema de salud que debe ser atendido de manera integral, promoviendo una alimentación balanceada y un estilo de vida saludable; con la participación de las autoridades, la industria, las organizaciones internacionales, la sociedad civil y la población en general. |  | <https://www.panamaamerica.com.pa/economia/impuesto-las-bebidas-azucaradas-crea-polemica-1104131> | 03-ago-20 |
| P17 | La Doña | Panamá América | Direct involvement and influence in policy | Costs Threats | "Cualquier nueva medida impositiva repercutirá negativamente en el agro, la industria y consecuentemente en la economía del país, por lo que el SIP se opone rotundamente a esta iniciativa legislativa" [Ley 570], señaló la SIP. Agregó que esta medida puede tener un efecto sobre la inversión y los más de 24 mil empleos directos e indirectos que dependen de esta industria. De acuerdo con el sindicato, la industria de bebidas azucaradas se ha visto afectada por las importaciones en los últimos años. Además ha decrecido de manera desmedida y esta medida afectaría la competitividad de los productores. |  | <https://www.panamaamerica.com.pa/economia/impuesto-las-bebidas-azucaradas-crea-polemica-1104131> | 03-ago-20 |
| P18 | Industrial Labor Union of Panama | Twitter | Discursive strategy | Frame the debate | Sindicato de Industriales de Panamá @industrialespty Como gremio responsable y preocupado por el bienestar y salud de la población manifestamos nuestra posición sobre la reciente Ley 114 de 18 de noviembre de 2019. #ValorandoLaIndustriaNacional El Sindicato de Industriales de Panamá(SIP), gremio que representa, defiende y promueve el desarrollo de la Industria Nacional, manifestamos nuestra posición sobre la reciente Ley 114 de 18 de noviembre de 2019, que crea el Plan de Acción para mejorar la Salud y dicta otras disposiciones para establecer el impuesto selectivo al consumo de bebidas azucaradas y los criterios para su uso. Como gremio responsable y preocupado por el bienstar y la salud de nuestra población, manifestamos nuestro interés de colaborar con el gobierno, la sociedad civil y otros actores, a través de medidas integrales que fomenten estilos de vida saludables combinados con una adecuada alimentación y actividad física. Sin embargo; desde la industria no compartimos el enfoque de emplear medidas fiscales discriminatorias, no obstante, reconocemos que, a diferencia de lo realizado en otros países en este caso, se trata de una medida que promueve la reformulación para ofrecer productos con menos contenido calórico a los consumidores. Queremos resaltar que para el sector industrial es importante crear ventajas comparativas con otros mercados y que seguiremos trabajando para brindar diferentes opciones que satisfagan las necesidades de los consumidores y sus hábitos de consumo. |  | <https://twitter.com/industrialespty/status/1197618307928596481> | 08-may-20 |
| P19 | Industrial Labor Union of Panama | La Prensa | Direct involvement and influence in policy Discursive strategy | Costs Threats | Impuesto a las bebidas azucaradas crea polémica. (...)Tras la aprobación de este proyecto, el Sindicato de Industriales de Panamá (SIP) informó que se opone a los proyectos de ley que creen o incrementen los impuestos a las empresas y consumidores, ya que coloca a la industria en desventaja competitiva con otros países de la región, con las consecuencias de la reducción de la inversión industrial y la posible pérdida de empleos. |  | <https://www.prensa.com/impresa/panorama/Impuesto-bebidas-azucaradas-crea-polemica_0_5242725759.html> | 03-ago-20 |
| P20 | Industrial Labor Union of Panama | La Prensa | Direct involvement and influence in policy | Direct actors | Impuesto a las bebidas azucaradas crea polémica. (...) Además, planteó que junto con la Cámara de Comercio, Industrias y Agricultura de Panamá participaron en el primer debate del proyecto, pero sus sugerencias no fueron tomadas en cuenta. |  | <https://www.prensa.com/impresa/panorama/Impuesto-bebidas-azucaradas-crea-polemica_0_5242725759.html> | 03-ago-20 |
| P21 | Industrial Labor Union of Panama | La Prensa | Discursive strategy | Frame the debate | El SIP opinó que difícilmente esta ley solucione los problemas de salud en el país. “La salud es afectada por multifactores, como malos hábitos de alimentación, sedentarismo, genética, entre otros”, acotó. Para la organización, la forma de abordar el problema es desde muy temprana edad, con educación y promoviendo buenos hábitos de alimentación y actividad física. |  | <https://www.prensa.com/impresa/panorama/Impuesto-bebidas-azucaradas-crea-polemica_0_5242725759.html> | 03-ago-20 |
| P22 | Industrial Labor Union of Panama | La Prensa | Direct involvement and influence in policy | Direct actors | Los señalamientos del SIP son respaldados por la Cámara de Comercio, Industrias y Agricultura de Panamá, que plantea que formará parte de la comisión diseñada para el mejoramiento de la salud, que verificará el destino final de los fondos producto de esta ley. “Si bien es cierto fuimos consultados, estimamos que no se terminó de evaluar y considerar a profundidad el impacto directo que esto tendría sobre los sectores involucrados en el tema, ni los efectos colaterales de la aprobación de leyes como esta”, expresó el gremio. |  | <https://www.prensa.com/impresa/panorama/Impuesto-bebidas-azucaradas-crea-polemica_0_5242725759.html> | 03-ago-20 |
| P23 | Industrial Labor Union of Panama | La Prensa | Discursive strategy | Frame the debate | Respecto al tema, el viceministro de Salud, Eric Ulloa, coincidió con los empresarios. Expresó que las medidas impositivas por sí solas no cambian los hábitos de consumo de las personas. “En el caso del consumo de cigarrillos, por ejemplo, las clínicas de cesación de tabaco han ayudado para que los pacientes dejen de fumar. Mientras que con el consumo de bebidas con exceso de azúcar la clave es la educación en el hogar y las escuelas”, manifestó. |  | <https://www.prensa.com/impresa/panorama/Impuesto-bebidas-azucaradas-crea-polemica_0_5242725759.html> | 03-ago-20 |
| P24 | Industrial Labor Union of Panama | Centralamericadata.com | Discursive strategy | Frame the debate | Nuevo impuesto a las bebidas azucaradas. Anel Flores, directivo de la Central Azucarera de Alanje, dijo a Laestrella.com.pa que "... se trata de una medida que ‘realmente no va a contribuir a la reducción de la obesidad y la diabetes'. La solución no es ‘satanizar el azúcar como culpable de la obesidad y del tema de la diabetes, es muy exagerado'." |  | <https://www.centralamericadata.com/es/article/home/Nuevo_impuesto_a_las_bebidas_azucaradas> | 04-ago-20 |
| P25 | Industrial Labor Union of Panama | Centralamericadata.com | Discursive strategy | Costs | Nuevo impuesto a las bebidas azucaradas. Flores añadió que "... el proyecto de ley podría afectar a una industria azucarera que emplea directa e indirectamente a 25 mil individuos, al igual que a los diferentes productores de jugos y gaseosas. ‘Eso va a encarecer al producto, afectando al consumidor final; va a traer reducción de personal en todas esas plantas', |  | <https://www.centralamericadata.com/es/article/home/Nuevo_impuesto_a_las_bebidas_azucaradas> | 04-ago-20 |
| P26 | Industrial Labor Union of Panama | Centralamericadata.com | Direct involvement and influence in policy | Indirect access-lobbying | Nuevo impuesto a las bebidas azucaradas (...)además recomiendo al presidente Juan Carlos Varela vetar el proyecto de ley, al considerar que no es conveniente, dada la situación económica actual, adoptar nuevos tributos e impuestos que van a atentar contra ‘el sector más vulnerable, que es el de la empleomanía rural y agrícola del país'." |  | <https://www.centralamericadata.com/es/article/home/Nuevo_impuesto_a_las_bebidas_azucaradas> | 04-ago-20 |
