# Supplementary material for: Two countries, similar practices: the political practices of the food industry influencing the adoption of key public health nutrition policies in Guatemala and Panama
Source: Public Health Nutr. 2022 Aug 22;25(11):3252–64. doi: 10.1017/S1368980022001811 (PMC9991768; doi:10.1017/S1368980022001811)
Supplement: Supplementary file 1 [file S1368980022001811sup.zip › S1368980022001811sup001.docx]

Supplemental Material 1S. Corporate Political Activity from publicly available data by food industry actor in Guatemala (2017-2020)

| Code | Food indusry actor | Source | Strategy | Code | Data coded | Notes | Website URL | Date collected |
| --- | --- | --- | --- | --- | --- | --- | --- | --- |
| G1 | Interamerican Pepsicola of Guatemala | Website | Direct involvement and influence in policy  Discursive strategies | Development and promotion of public-private initiatives and self-regulation Frame the debate | CBC cumple y respeta las leyes y reglamentos de etiquetado de todos los países en los que opera. Así mismo, nuestros socios estratégicos PepsiCo y BELIV, tomaron la decisión de colocar en la parte frontal del etiquetado el contenido calórico de los productos, inclusive en los países en donde la legislación no lo solicita. | Comunicación en Progreso 2019 Pacto Global de las Naciones Unidas Pág. 63 | <https://cbc.co/wp-content/uploads/2019/ComunicacionProgresocbc2019.pdf> | 22-abr-20 |
| G2 | Interamerican Pepsicola of Guatemala | Website | Discursive strategies | Frame the debate | Buscamos que nuestros consumidores tomen decisiones informadas para ellos y sus familias, por esa razón incluimos información clara sobre los ingredientes de nuestros productos en las etiquetas. | Comunicación en Progreso 2019 Pacto Global de las Naciones Unidas Pág. 63 | <https://cbc.co/wp-content/uploads/2019/ComunicacionProgresocbc2019.pdf> | 22-abr-20 |
| G3 | Interamerican Pepsicola of Guatemala | Website | Direct involvement and influence in policy | Development and promotion of public-private initiatives and self-regulation | Los avances de Beliv en materia de etiquetado son los siguientes: el 70% de los productos cuentan con GDAs, Guideline Daily Amounts, un 15% está en proceso de implementación y el 15% restante, está adaptado a las regulaciones locales. | Comunicación en Progreso 2019 Pacto Global de las Naciones Unidas Pág. 63 | <https://cbc.co/wp-content/uploads/2019/ComunicacionProgresocbc2019.pdf> | 22-abr-20 |
| G4 | Coca Cola of Guatemala S.A. | Website | Discursive strategies | Frame the debate | Dada la posición de nuestra compañía en Latinoamérica, nuestras etiquetas nutricionales reconocen que cada población es distinta, con sus propias necesidades y hábitos. | Reporte Anual 2019 Pág. 16 | <https://img.coca-colafemsa.com/assets/files/es/inversionistas/Coca-Cola-FEMSA-Informe-Integrado-2019.pdf> | 22-abr-20 |
| G5 | Coca Cola of Guatemala S.A. | Website | Direct involvement and influence in policy  Discursive strategies | Development and promotion of public-private initiatives and self-regulation Frame the debate | Por lo tanto, respaldamos y cumplimos con el marco legal existente de cada país. Cuando surgen cambios de regulación, siempre estamos dispuestos a participar proactivamente proporcionando nuestra experiencia e información de calidad para asegurar que los clientes reciban información de alta calidad. Además, nuestro proceso de producción cumple con los estándares más altos de calidad, y nuestros ingredientes en todas nuestras operaciones cumplen con las regulaciones locales y con los altos estándares internos de Coca-Cola. | Reporte Anual 2019 Pág. 16 | <https://img.coca-colafemsa.com/assets/files/es/inversionistas/Coca-Cola-FEMSA-Informe-Integrado-2019.pdf> | 22-abr-20 |
| G6 | Coca Cola of Guatemala S.A. | Website | Discursive strategies | Frame the debate | Decisiones nutricionales informadas: Para permitir que los consumidores tomen decisiones nutricionales informadas, las etiquetas de nuestros productos cuentan información clara y fácil de usar, incluyendo, grasas, azúcares y sodio para cada uno de nuestros productos. | Reporte Anual  Pág. 46 | <https://img.coca-colafemsa.com/assets/files/es/inversionistas/Coca-Cola-FEMSA-Informe-Integrado-2019.pdf> | 22-abr-20 |
| G7 | Coca Cola of Guatemala S.A. | Website | Direct involvement and influence in policy | Development and promotion of public-private initiatives and self-regulation | Nuestro etiquetado nutricional tiene por objetivo proporcionar información clara y completa de acuerdo con las regulaciones aplicables en los 9 países donde operamos. Cuando existen cambios en la regulación, adaptamos el etiquetado con el fin de asegurar que nuestros consumidores obtengan información de alta calidad. |  | <https://img.coca-colafemsa.com/assets/files/es/inversionistas/Coca-Cola-FEMSA-Informe-Integrado-2019.pdf> | 22-abr-20 |
| G8 | Nestlé S.A. | Website | Discursive strategies | Frame the debate | Nestlé®, a través del etiquetado de sus envases, provee información clara sobre la cantidad de energía, azúcar, sal, grasas, fibra y proteínas que contienen sus productos, así como una tabla con todas las vitaminas y los minerales que proporcionan los productos que comercializa la compañía. |  | [https://www.nestle-centroamerica.com/featuredstories/nestle-lanza-campana-para-educar-sobre-el-consumo-de-cereales-para-el-desayuno0522-7825](https://www.nestle-centroamerica.com/featuredstories/nestle-lanza-campana-para-educar-sobre-el-consumo-de-cereales-para-el-desayuno0522-7823) | 08-may-20 |
| G9 | Bimbo | Facebook | Direct involvement and influence in policy  Discursive strategies | Development and promotion of public-private initiatives and self-regulation Frame the debate | POLÍTICA GLOBAL DE ETIQUETADO Con el fin de asegurar, a nivel Grupo Bimbo, el cumplimiento de la regulación aplicable al etiquetado nutrimental y de definir el mínimo de información que debemos proporcionar a nuestros consumidores sobre el tema, es política de Grupo Bimbo cumplir con los siguientes lineamientos: · Cumplir cabalmente con la legislación local en materia de etiquetado nutrimental. · Incorporar en todos nuestros productos, como mínimo y adicional a la información nutrimental obligatoria. · Incorporar en todos nuestros productos, adicional a la información nutrimental básica, un etiquetado frontal nutrimental sencillo y comprensible. · Contar con sustento científico sólido para todas las declaraciones de propiedad saludables. · Cumplir íntegramente con las regulaciones definidas a nivel local sobre porciones recomendadas | Estrategia 2020 | <https://grupobimbo.com/sites/default/files/Grupo-Bimbo-Estrategia-Salud-y-bienestar.pdf> | 08-may-20 |
| G10 | Bimbo | Facebook | Discursive strategies | Frame the debate | La tendencia de preferencia entre Los consumidores por información de ingredientes fácil de entender y transparente ha incrementado. porque consideran que la comida debe cumplir con la promesa de ser simple. clara. confiable. transparente y auténtica. En GB estamos comprometidos a reformular o lanzar productos con ingredientes amigables en La Lectura de la etiqueta y una sólida historia de sustentabilidad para ser La principal opción de nuestros consumidores. Los Lineamientos nutricionales de GB también consideran evaluarla calidad de La composición de nuestras recetas a través de una estrategia de etiqueta Limpia. | GB Lineamientos Nutricionales 2019 | <https://grupobimbo.com/sites/default/files/Grupo-Bimbo-Guia-Lineamientos.pdf> | 08-may-20 |
| G11 | Guatemalan Chamber of Industry | Twitter | Coalition Management | Intern | Conversación Cámara de Industria de Guatemala @industriaguate En el Foro de Perspectivas Regulatorias Internacionales se aborda el tema: El escenario en América Latina y su impacto en la reputación de los alimentos procesados.  @CORPCMI   @Gremabgt   \|  \| \| --- \| |  | <https://twitter.com/industriaguate/status/1217544866122686464/photo/1> | 08-may-20 |
| G12 | CGAB-Guatemalan Chamber of Food and Beverages | Website | Coalition Management | Intern | El Comité de Etiquetado tiene como fin analizar las iniciativas de ley, acuerdos gubernativos y reglamentos nacionales e internacionales que involucren el etiquetado de Alimentos y Bebidas. |  | <http://cgab.org.gt/index.php/comites> | 08-may-20 |
| G13 | CGAB-Guatemalan Chamber of Food and Beverages | Website | Discursive strategies Information management | Frame the debate Amplification | Mito #1 Los alimentos procesados provocan hipertensión, cáncer diabetes, entre otras enfermedades Realidad Alimentación no balanceada Insuficiente actividad física Factores genéticos, ambientales o culturales Malos hábitos de consumo | CGAB en acción Boletín 9 Septiembre 2018 pág. 3 | <http://cgab.org.gt/images/investigaciones/Boletn-septiembre-CGAB.pdf> | 08-may-20 |
| G14 | CGAB-Guatemalan Chamber of Food and Beverages | Website | Coalition Management | Intern | En la reunión de la Coalición Centroamericana de Alimentos realizada en San Salvador el 6 de septiembre, participó el Ing. Enrique Lacs, Director Ejecutivo de la CGAB junto con todas las asociaciones de los 7 países de Centro América, para revisar los avances del CODEX Alimentarius y las iniciativas regionales sobre etiquetado frontal. | CGAB en acción Boletín 9 Septiembre 2018 pág. 4 | <http://cgab.org.gt/images/investigaciones/Boletn-septiembre-CGAB.pdf> | 08-may-20 |
| G15 | CGAB-Guatemalan Chamber of Food and Beverages | Website | Information management | Suppression | El argumento de los diputados es que con esta ley se ataca “la comida chatarra”, ya que establece límites máximos de nutrientes para poder comercializar los alimentos procesados. Es importante reconocer que con esta iniciativa los diputados han declarado como comida chatarra al tamal, pache, dulces típicos, chuchitos, chicharrones, chojín etc., puesto que ninguno de estos cumple con los límites de nutrientes y por tanto serían también responsables directos de las enfermedades no transmisibles. | CGAB en acción Boletín 11 Octubre 2018 pág. 1 | <http://cgab.org.gt/images/investigaciones/Boletn-segunda-quincena-octubre-CGAB.pdf> | 08-may-20 |
| G16 | CGAB-Guatemalan Chamber of Food and Beverages | Website | Discursive strategies | Costs Frame the debate | Al superar los límites de nutrientes, las empresas estarían obligadas a colocar tantos sellos de advertencia en sus etiquetas, como límites rebasados por nutriente. La forma de evitar el consumo “alto” de estos nutrientes es castigar con más costos a los productores formales de alimentos y bebidas con un nuevo impuesto encubierto en forma de una tasa de autorización de etiquetas, que va de 10% por un sello hasta 35% por los 5 sellos posibles. | CGAB en acción Boletín 11 Octubre 2018 pág. 1 | <http://cgab.org.gt/images/investigaciones/Boletn-segunda-quincena-octubre-CGAB.pdf> | 08-may-20 |
| G17 | CGAB-Guatemalan Chamber of Food and Beverages | Website | Discursive strategies | Good governance  Frame the debate | Codex Alimentarius elabora una guía para el Etiquetado Frontal Nutricional -EFN Asimismo, establece que la elaboración del sistema EFN debe ser liderada por el gobierno, pero desarrollada en colaboración con todas las partes interesadas, incluyendo sector privado, consumidores, la academia, entre otros. La CGAB participa en la discusión para aportar observaciones a la guía del Codex. La CGAB considera que es necesario que el consumidor esté bien informado del contenido de los alimentos y de sus nutrientes. Este tipo de regulaciones deben de realizarse a nivel regional mediante un reglamento técnico centroamericano, para preservar la zona de libre comercio centroamericana | CGAB en acción Boletín 17 Febrero 2019 pág. 1 | <http://cgab.org.gt/images/investigaciones/Boletn--primera-quincena--febrero-2019.pdf> | 08-may-20 |
| G18 | CGAB-Guatemalan Chamber of Food and Beverages | Website | Information management | Amplification | Apoyamos que cualquier regulación para este tipo de etiquetado se base en lo establecido por CODEX y que el sistema de EFAN adecuado debe dar la información al consumidor según las Guías Diarias Alimentarias (GDA) con las cantidades diarias de ingredientes a consumir. El sello o ícono monocromático debe favorecer la fácil comprensión del consumidor en el momento de la compra del producto. | CGAB en acción Boletín 17 Febrero 2019 pág. 1 | <http://cgab.org.gt/images/investigaciones/Boletn--primera-quincena--febrero-2019.pdf> | 08-may-20 |
| G19 | CGAB-Guatemalan Chamber of Food and Beverages | Website | Information management | Amplification | Comprendiendo la etiqueta nutricional La etiqueta de información nutricional debe estar en idioma español. Los productos con etiquetas en otros idiomas deben tener una etiqueta pegada en idioma español. Las etiquetas deben ser legibles, colocadas de forma accesible y contener la información que establece la norma centroamericana. | CGAB en acción Boletín 23 Mayo 2019 pág. 4 | <http://cgab.org.gt/images/investigaciones/Boletn--primera-quincena-de-mayo-de-2019.pdf> | 08-may-20 |
| G20 | CGAB-Guatemalan Chamber of Food and Beverages | Website | Information management | Suppression | Por otro lado, aunque la CGAB comparte el objetivo de prevenir las enfermedades no transmisibles considera que la iniciativa de ley será ineficaz ya que no toma en cuenta los hábitos alimenticios y la forma de acceso de los alimentos de la población guatemalteca | CGAB en acción Boletín 10 Octubre 2018 pág. 1 | <http://cgab.org.gt/images/investigaciones/Boletn-primera-quincea-octubre-CGAB.pdf> | 08-may-20 |
| G21 | CGAB-Guatemalan Chamber of Food and Beverages | Website | Coalition Management | Intern | Este evento será un espacio que reunirá a los protagonistas de este sector productor, la cual contará con tres actividades: 10 conferencias en diferentes temas sobre etiquetado, impuestos, innovación, medio ambiente, un foro que tratará sobre la Importancia Impacto de la Unión Aduanera y la rueda de negocios que contará con la participación de importantes empresas internacionales, brindando la oportunidad de realizar negociaciones estratégicas de alto nivel | CGAB en acción Boletín 10 Octubre 2018 pág. 2 | <http://cgab.org.gt/images/investigaciones/Boletn-primera-quincea-octubre-CGAB.pdf> | 08-may-20 |
| G22 | CGAB-Guatemalan Chamber of Food and Beverages | Website | Information management | Amplification | La CGAB participó en el Conversatorio Iniciativa 5504 Ley de Promoción de Alimentación Saludable que organizó el Colegio de Químicos Farmacéuticos con el apoyo de la Asociación de Nutricionistas de Guatemala. El conversatorio contó con la participación de la asesora del diputado Jairo Flores, la Dra. Fernanda Kroker (INCAP), Msc. Mair Ruano (SESAN), Dr. Adrián Chávez (IPNUSAC) | CGAB en acción Boletín 16 Enero 2018 pág. 4 | <http://cgab.org.gt/images/investigaciones/Boletn-segunda-quincena--enero-2019.pdf> | 08-may-20 |
| G23 | CGAB-Guatemalan Chamber of Food and Beverages | Website | Coalition Management | Intern | La Alianza Latinoamericana de Asociaciones de la Industria de Alimentos y Bebidas se reunió en Colombia para tratar temas comunes para sus miembros como las normas de etiquetado, publicidad, impuestos y el aporte de los alimentos y bebidas al cumplimiento de los Objetivos de Desarrollo Sostenible (ODS). | CGAB en acción Boletín 24 Mayo 2019 pág. 4 | <http://cgab.org.gt/images/investigaciones/Boletn-segunda-quincena-de-mayo-de-2019.pdf> | 08-may-20 |
| G24 | CGAB-Guatemalan Chamber of Food and Beverages | Website | Information management Discursive strategies | Amplification Frame the debate | La Cámara Guatemalteca de Alimentos y Bebidas (CGAB) está en desacuerdo con cinco iniciativas de ley que modificarían las normativas para el etiquetado de productos y el aumento del Impuesto al Valor Agregado (IVA) de 12 a 20 por ciento a las bebidas azucaradas. “Estamos de acuerdo en que se ofrezca información objetiva al consumidor en la etiqueta, pero no colocar un círculo rojo o una equis por la cantidad de azúcar que contiene”, indicó Enrique Lacs, director ejecutivo de la CGAB. | <https://elperiodico.com.gt/inversion/2018/05/30/rechazan-iniciativas-de-etiquetado/> | <http://cgab.org.gt/index.php/noticias/prensa/197-rechazan-iniciativas-de-etiquetado> | 08-may-20 |
| G25 | CGAB-Guatemalan Chamber of Food and Beverages | Website | Information management | Suppression | El paquete de normativas estarían amparadas en la salud de la población, pero según Lacs, están basadas en desinformación, y lo que ocasionarían es un aumento de impuestos, que elevaría el precio del producto y la canasta básica.  “Los consumidores buscarían un sustituto más barato o adquirir el mismo de otra fuente, el contrabando es una de ellas”, explicó. | <https://elperiodico.com.gt/inversion/2018/05/30/rechazan-iniciativas-de-etiquetado/> | <http://cgab.org.gt/index.php/noticias/prensa/197-rechazan-iniciativas-de-etiquetado> | 08-may-20 |
| G26 | CGAB-Guatemalan Chamber of Food and Beverages | Website | Information management | Suppression | Manuel Torres, director de la cámara empresarial, manifestó que hay una serie de publicaciones sin soporte científico que pretenden responsabilizar a los alimentos procesados de provocar enfermedades. | <https://elperiodico.com.gt/inversion/2018/05/30/rechazan-iniciativas-de-etiquetado/> | <http://cgab.org.gt/index.php/noticias/prensa/197-rechazan-iniciativas-de-etiquetado> | 08-may-20 |
| G27 | CGAB-Guatemalan Chamber of Food and Beverages | Website | Information management Discursive strategies | Suppression  Frame the debate | Los empresarios coincidieron que hay publicaciones sobre los alimentos conservados que pueden ocasionar un efecto negativo en la industria alimenticia al confundir al consumidor, por lo que lanzaron la campaña “Vida Saludable”. | <https://elperiodico.com.gt/inversion/2018/05/30/rechazan-iniciativas-de-etiquetado/> | <http://cgab.org.gt/index.php/noticias/prensa/197-rechazan-iniciativas-de-etiquetado> | 08-may-20 |
| G28 | CGAB-Guatemalan Chamber of Food and Beverages s | Website | Information management Discursive strategies | Suppression  Frame the debate | Enrique Lacs, director ejecutivo de la CGAB, informó que a través de la campaña [Vida Saludable] se busca ofrecer a la población información objetiva sobre los alimentos que procesan, “existe mala información sobre los productos procesados al decir que causan cáncer”. | <https://emisorasunidas.com/2018/05/29/camara-guatemalteca-de-alimentos-lanza-campana-vida-saludable/> | <http://cgab.org.gt/index.php/noticias/prensa/196-camara-guatemalteca-de-alimentos-lanza-campana-vida-saludable> | 08-may-20 |
| G29 | CGAB-Guatemalan Chamber of Food and Beverages | Website | Information management Discursive strategies | Suppression  Frame the debate | Lanzan campaña para evitar desinformación sobre productos procesados La Cámara Guatemalteca de Alimentos y Bebidas (CGAB) lanzó hoy una campaña denominada “Vida Saludable”, la cual tiene como fin aclarar toda la desinformación que se genera en torno a los alimentos procesados. La campaña [Vida Saludable] se da en medio de varias iniciativas impulsadas en el Congreso de la República que buscan aumentar los controles sobre este tipo de alimentos, señalados de ser responsables de enfermedades como la diabetes y el cáncer, entre otras. La Cámara considera que esas leyes impulsadas en el Legislativo son oportunistas y que tienen distintos intereses específicos. Además, señalan que se están amparando en la salud de los guatemaltecos para conseguir esos objetivos. | <http://cgab.org.gt/index.php/noticias/prensa/194-lanzan-campana-para-evitar-desinformacion-sobre-productos-procesados> | <https://lahora.gt/lanzan-campana-para-evitar-desinformacion-sobre-productos-procesados/> | 08-may-20 |
| G30 | CGAB-Guatemalan Chamber of Food and Beverages | Website | Discursive strategies | Frame the debate | Enrique Lacs, director ejecutivo de la Cámara, indicó que con esta campaña [Vida Saludable] no se está buscando evitar un aumento en los impuestos, sino generar una concientización sobre los alimentos que se consumen. Lacs indicó también que, en Guatemala solo un 25 por ciento de los alimentos que consumen en los hogares son procesados | <http://cgab.org.gt/index.php/noticias/prensa/194-lanzan-campana-para-evitar-desinformacion-sobre-productos-procesados> | <https://lahora.gt/lanzan-campana-para-evitar-desinformacion-sobre-productos-procesados/> | 08-may-20 |
| G31 | CGAB-Guatemalan Chamber of Food and Beverages | Website | Direct involvement and influence in policy | Development and promotion of public-private initiatives and self-regulation | La propuesta pretende implementar sellos de advertencia nutricional en empaques de los productos para que el consumidor sepa si lo que compra puede dañar su salud, algo que la Cámara Guatemalteca de Alimentos y Bebidas (CGAB), considera que incrementará el precio de la canasta básica, además de ser innecesaria, ya que la industria cumple con normativas sobre cantidades de nutrientes. | <https://www.soy502.com/articulo/empresarios-impuesto-comida-chatarra-amenaza-economia-32539> | <http://cgab.org.gt/index.php/noticias/prensa/189-empresarios-impuesto-a-comida-chatarra-amenaza-la-economia> | 08-may-20 |
| G32 | CGAB-Guatemalan Chamber of Food and Beverages | Website | Discursive strategies | Costs | “El anteproyecto de Ley afecta en primer lugar al consumidor, luego al productor nacional y al importador porque obliga a reformular muchos de los productos que están en el mercado, lo cual requiere inversión, capacidad técnica y tiempo”explica Enrique Lacs, Directos Ejecutivo de la CGAB | [https://www.soy502.com/articulo/empresarios-impuesto-comida-chatarra-amenaza-economia-32539  La información se encontró nuevamente en el Facebook https://www.facebook.com/photo?fbid=311859249395922&set=pcb.311859332729247](https://www.soy502.com/articulo/empresarios-impuesto-comida-chatarra-amenaza-economia-32539) | <http://cgab.org.gt/index.php/noticias/prensa/189-empresarios-impuesto-a-comida-chatarra-amenaza-la-economia> | 08-may-20 |
| G33 | CGAB-Guatemalan Chamber of Food and Beverages | Website | Discursive strategies | Costs | Además, la CGAB ve como otra amenaza de la iniciativa, el impacto que esta tendrá en las importaciones de los productos que no podrían cumplir con el reglamento del nuevo etiquetado. | <https://www.soy502.com/articulo/empresarios-impuesto-comida-chatarra-amenaza-economia-32539> | <http://cgab.org.gt/index.php/noticias/prensa/189-empresarios-impuesto-a-comida-chatarra-amenaza-la-economia> | 08-may-20 |
| G34 | CGAB-Guatemalan Chamber of Food and Beverages | Website | Direct involvement and influence in policy | Development and promotion of public-private initiatives and self-regulation | La Ley de Promoción de Alimentación Saludable propuesta, incrementaría el precio de la canasta básica, siendo innecesaria, ya que la industria de bebidas y alimentos cumple con normativas nacionales e internacionales referentes a las cantidades de nutrientes y etiquetado. | <http://guatenews.com/noticias/proponen-impuesto-la-comida-rapida/> | <http://cgab.org.gt/index.php/noticias/prensa/186-proponen-un-impuesto-para-la-comida-rapida> | 08-may-20 |
| G35 | CGAB-Guatemalan Chamber of Food and Beverages | Website | Coalition management | Fragmentation and destabilization | Empresarios del sector de alimentos y bebidas consideran que la propuesta de Ley de Promoción de Alimentación Saludable, promovida por la Comisión de Desarrollo Social, presidida por el diputado Jairo Flores, del partido de la Unidad Nacional de la Esperanza, y otros diputados, es preocupante debido a que pretende establecer nuevos impuestos a los alimentos y bebidas. Este nuevo impuesto busca eliminar la obesidad y las enfermedades que genera este estado | <http://guatenews.com/noticias/proponen-impuesto-la-comida-rapida/> | <http://cgab.org.gt/index.php/noticias/prensa/186-proponen-un-impuesto-para-la-comida-rapida> | 21-may-20 |
| G36 | CGAB-Guatemalan Chamber of Food and Beverages | Website | Information management | Amplification | Exige que los alimentos procesados nacionales e importados se etiquete el producto colocando un sello por cada ingrediente que sobrepase la cantidad límite establecida para cada alimento. Actualmente ya existe legislación internacional de etiquetado que indica con claridad las cantidades de los ingredientes que contiene cada producto, e informa al consumidor lo que está comprando. | <http://guatenews.com/noticias/proponen-impuesto-la-comida-rapida/> | <http://cgab.org.gt/index.php/noticias/prensa/186-proponen-un-impuesto-para-la-comida-rapida> | 21-may-20 |
| G37 | CGAB-Guatemalan Chamber of Food and Beverages | Website | Information management | Amplification | El nuevo impuesto se aplicaría en forma directa a la cantidad de etiquetas en forma de sellos de advertencia sobre el exceso de cada ingrediente. El rango del nivel impositivo va desde 5% hasta 35% sobre el precio final del producto al consumidor. Este impuesto causará un grave impacto en la económica familiar, y no necesariamente resolverá la obesidad de la población. | [La información se encontró de nuevo en el Website y Facebook de la Cámara guatemalteca de alimentos y bebidas y en CGAB en acción Boletín 10 Octubre 2018 pág. 1 http://guatenews.com/noticias/proponen-impuesto-la-comida-rapida/ http://cgab.org.gt/index.php/noticias/prensa/175-la-iniciativa-de-ley-que-confronta-a-diputados-y-la-cgab https://www.facebook.com/photo?fbid=311859249395922&set=pcb.311859332729247 https://www.soy502.com/articulo/empresarios-impuesto-comida-chatarra-amenaza-economia-32539 http://cgab.org.gt/images/investigaciones/Boletn-primera-quincea-octubre-CGAB.pdf](http://guatenews.com/noticias/proponen-impuesto-la-comida-rapida/La%20información%20se%20encontró%20de%20nuevo%20en%20el%20Website%20y%20Facebook%20de%20la%20Cámara%20guatemalteca%20de%20alimentos%20y%20bebidas) | <http://cgab.org.gt/index.php/noticias/prensa/186-proponen-un-impuesto-para-la-comida-rapida> | 22-may-20 |
| G38 | CGAB-Guatemalan Chamber of Food and Beverages | Website | Direct involvement and influence in policy  Information management | Development and promotion of public-private initiatives and self-regulation  Amplification | Según el diputado Flores la iniciativa en mención vendría a proteger el derecho a la salud y su fin es reducir las enfermedades crónicas y no transmisibles. Para Enrique Lacs, el tema de salud se deriva de los malos hábitos alimenticios, los excesos del consumo y la falta de ejercicio y con impuestos no se resolverá. “Creemos que esta iniciativa no es necesaria y que no ha sido analizada debidamente. Los productores nacionales cumplimos con las normativas nacionales e internacionales referentes a las cantidades de nutrientes y etiquetado. Como CGAB estamos comprometidos todo el tiempo a brindar información que ayude a las personas a tomar decisiones más acertadas para el bienestar de las personas”, finalizó. | http://guatenews.com/noticias/proponen-impuesto-la-comida-rapida/  La información se encontró nuevamente en el Facebook de la Cámara guatemalteca de alimentos y bebidas https://www.facebook.com/photo?fbid=311859249395922&set=pcb.311859332729247 | http://cgab.org.gt/index.php/noticias/prensa/186-proponen-un-impuesto-para-la-comida-rapida | 22-may-20 |
| G39 | CGAB-Guatemalan Chamber of Food and Beverages | Website | Information management Discursive strategy | Amplification Frame the debate | La iniciativa de ley de promoción de alimentación saludable que busca se coloque un sello de advertencia nutricional al frente de cada empaque o lata llegó al Congreso. No todos apoyan la propuesta.  La Cámara Guatemalteca de Alimentos y Bebidas (CGAB) considera que la propuesta de ley, promovida por la Comisión de Desarrollo Social, como una amenaza directa a la producción, al comercio y a la economía del país. | <http://cgab.org.gt/index.php/noticias/prensa/175-la-iniciativa-de-ley-que-confronta-a-diputados-y-la-cgab> | <https://republica.gt/2018/10/07/iniciativa-ley-confronta-diputados-cgab/> | 22-may-20 |
| G40 | CGAB-Guatemalan Chamber of Food and Beverages | Facebook | Discursive strategy | Frame the debate | La CGAB busca aclarar el acceso a la información nutricional que el consumidor necesita saber sobre la composición de los alimentos. Organismos internacionales, ONGs, y varios políticos consideran que estas etiquetas (EFAN) le dan mejor información sobre los alimentos. ¿Cree que cuenta con toda la información que necesita? |  | <https://www.facebook.com/photo/?fbid=627175774530933&set=a.238559240059257> | 22-may-20 |
| G41 | CGAB-Guatemalan Chamber of Food and Beverages | Facebook | Information management | Suppression | ¿Cuál de estos dos métodos de etiquetado le da mejor información del producto? (Imagen que por un lado muestra los octágonos de advertencia nutricional: alto en azúcares, alto en grasas saturadas, alto en sodio, alto en calorías e invita a la comparación con la tabla de Información Nutricional con las cantidades de los nutrientes) ¿Con sellos negros de etiquetado ALTO EN, usted puede determinar las cantidades de nutrientes en el producto? |  | <https://www.facebook.com/photo/?fbid=626523087929535&set=a.238559240059257> | 22-may-20 |
| G42 | CGAB-Guatemalan Chamber of Food and Beverages | Facebook | Information management | Suppression | De estos dos tipos de etiquetado ¿Cuál considera que le da más información? (EFAN vs. Etiqueta) (Imagen que por un lado muestra los octágonos de advertencia nutricional: alto en azúcares, alto en grasas saturadas, alto en sodio, alto en calorías e invita a la comparación con la tabla de Información Nutricional con las cantidades de los nutrientes) |  | <https://www.facebook.com/photo?fbid=624236461491531&set=a.238559240059257> | 22-may-20 |
| G43 | CGAB-Guatemalan Chamber of Food and Beverages | Facebook | Discursive strategy | Frame the debate | [Conoce la importancia de los nutrientes en el siguiente post. #vidaSaludable  La clave está en la cantidad de cada nutriente por cada porción, los cuales se detallan en las etiquetas de los alimentos procesados.](https://www.facebook.com/hashtag/vidasaludable?__cft__%5b0%5d=AZXovQ31N47XCmp8P1R0eVB6QKBCu-DLHgfdv7S4-WMa0CgQPoEcd4Q4q48bYHTISSCOgLlLbV89DwQIY1gRYcAn1VBtGWzULoj9Cw_flHXeY_-4VowhBztZbfLG-Chjw6_49ekT4Ql7mj-cRUpnrCUqLDIrYplJ_Wi8zC3-lREG7w&__tn__=*NK*F) | La información se encontró nuevamente en el twitter de la Cámara guatemalteca de alimentos y bebidas https://twitter.com/cgab2018/status/1149067470642339840/photo/1 | <https://www.facebook.com/photo/?fbid=443178502930662&set=a.238559240059257> | 22-may-20 |
| G44 | CGAB-Guatemalan Chamber of Food and Beverages | Facebook | Information management Discursive strategy | Amplification Frame the debate | Mito 3: Los ingredientes que contienen los alimentos procesados pueden perjudicar a las personas. Conoce la respuesta en el siguiente post. Mito#3: los ingredientes que contienen los alimentos procesados pueden perjudicar a las personas. Realidad: todos los alimentos procesados son inocuos y seguros para el consumo. Los ingredientes de los alimentos procesados son aprobados y autorizados por organismos nacionales e internacionales. La Organización Mundial de la Salud y el CODEX Alimentarius evalúan y autorizan los ingredientes, así como las cantidades apropiadas que deben utilizarse para el procesamiento de alimentos.  El procesamiento debe cumplir normas estrictas para asegurar su inocuidad. | La información se encontró nuevamente en el Website de la Cámara guatemalteca de alimentos y bebidas CGAB en acción Boletín 15 Enero 2019 pág. 5 http://cgab.org.gt/images/investigaciones/Boletn-primera-quincena--enero-2019.pdf | <https://www.facebook.com/photo/?fbid=441197589795420&set=a.238559240059257> | 22-may-20 |
| G45 | CGAB-Guatemalan Chamber of Food and Beverages | Facebook | Discursive strategy | Frame the debate | Mito #2: Los alimentos procesados no son tan nutritivos como los frescos. Realidad: Los alimentos procesados contribuyen a una nutrición balanceada. Los alimentos procesados son aprobados por el MSPAS para garantizar su valor nutricional antes de comercializarse. |  | <https://www.facebook.com/photo/?fbid=439014540013725&set=a.238559240059257> | 22-may-20 |
| G46 | CGAB-Guatemalan Chamber of Food and Beverages | Facebook | Information management | Amplification | La etiqueta nutricional tiene como objetivo informar al consumidor sobre las propiedades nutricionales de un alimento. #VidaSaludable los alimentos procesados envasados deben estar etiquetados. La etiqueta debe contener información nutricional.  Valor de referencia de nutriente (VRN): cantidad diaria de ingestión de energía o nutrientes por persona, establecidos por FAO/OMS. El etiquetado nutricional debe proporcionar al consumidos información acerca del tipo y cantidad de nutrientes aportados por el alimento. | La información se encontró nuevamente en el Facebook de la Cámara guatemalteca de alimentos y bebidas https://www.facebook.com/photo/?fbid=383497225565457&set=a.238559240059257 | <https://www.facebook.com/photo/?fbid=429927417589104&set=a.238559240059257> | 22-may-20 |
| G47 | CGAB-Guatemalan Chamber of Food and Beverages | Facebook | Information management | Amplification | ¿Sabía qué los alimentos procesados pueden mejorar algunas propiedades nutricionales de los alimentos frescos? Los alimentos procesados pueden mejorar algunas propiedades nutricionales de los alimentos frescos al agregárseles minerales (hierro, zinc, yodo, y otros) así como vitaminas (A, B, C, D, E), entre otros, de acuerdo con las normas nacionales e internacionales. |  | <https://www.facebook.com/photo/?fbid=421247378457108&set=a.238559240059257> | 22-may-20 |
| G48 | CGAB-Guatemalan Chamber of Food and Beverages | Facebook | Information management | Amplification | La clave está en la cantidad de cada nutriente por cada porción, los cuales se detallan en la etiqueta de alimentos procesados. |  | <https://www.facebook.com/photo/?fbid=417780432137136&set=a.238559240059257> | 26-may-20 |
| G49 | CGAB-Guatemalan Chamber of Food and Beverages | Facebook | Information management | Amplification | Esta es la etiqueta (tabla nutricional) que la ley vigente obliga a colocar en los productos y que contiene la información que requiere el consumidor. |  | <https://www.facebook.com/photo/?fbid=413567895891723&set=a.238559240059257> | 26-may-20 |
| G50 | CGAB-Guatemalan Chamber of Food and Beverages | Facebook | Information management | Amplification | Los estudios recientes en cuanto publicidad y salud muestran cómo la información contenida en un anuncio o en la etiqueta de un producto alimenticio, puede alterar la apreciación de su sabor. En esta perspectiva, la colocación de una etiqueta en los alimentos recomendados podría resultar efectiva si se acompaña de otras medidas que incorporan figuras y color. Los efectos contraproducentes identificados (la falta de placer del gusto presumido o la sensación de poder consumir estos productos a voluntad) podrían evitarse si dicha medida se asociara con una campaña de información efectiva simultánea y no solo una etiqueta. | [https://l.facebook.com/l.php?u=https%3A%2F%2Flahora.gt%2Fde-la-publicidad-a-la-salud-prevencion-de-la-obesidad%2F%3Ffbclid%3DIwAR0ohNXtcoJk23F742p8wHX-ZLbaV1nFQ0wpt4a9klh2sbGozdsEz1ySiyI&h=AT10edROTvOLV_sitzjlJ4p-dKx3vky4R3MWoos0gbdaVNHiZpGz9BLS-xDLY9pS96S6Sff42M61yDiQKsVl_IyiBuwwNmBN7pphq3IzrWarXXil_uhCrZHWves6AdykYg&__tn__=H-R&c[0]=AT3WbrPvS8bK2f2vSk6EPW4LCb5geWJRsccqbdVmBF6FHVA6gIubdjiXwulrcM6ZtRxiLCBpvwWXXnnSBPDkMg_2SJmzjQu268qALZvc1-M5PhWPnsQ4CKDto0b2V7tvmgZ7IM8r00aUI9EyOKwpJJGlwsH1Awqr38RgAWqgupKWYBwKwKJ_kdPYOMc](https://l.facebook.com/l.php?u=https%3A%2F%2Flahora.gt%2Fde-la-publicidad-a-la-salud-prevencion-de-la-obesidad%2F%3Ffbclid%3DIwAR0ohNXtcoJk23F742p8wHX-ZLbaV1nFQ0wpt4a9klh2sbGozdsEz1ySiyI&h=AT10edROTvOLV_sitzjlJ4p-dKx3vky4R3MWoos0gbdaVNHiZpGz9BLS-xDLY9pS96S6Sff42M61yDiQKsVl_IyiBuwwNmBN7pphq3IzrWarXXil_uhCrZHWves6AdykYg&__tn__=H-R&c%5b0%5d=AT3WbrPvS8bK2f2vSk6EPW4LCb5geWJRsccqbdVmBF6FHVA6gIubdjiXwulrcM6ZtRxiLCBpvwWXXnnSBPDkMg_2SJmzjQu268qALZvc1-M5PhWPnsQ4CKDto0b2V7tvmgZ7IM8r00aUI9EyOKwpJJGlwsH1Awqr38RgAWqgupKWYBwKwKJ_kdPYOMc) | <https://www.facebook.com/Vida-Saludable-GT-228946467687201/> | 26-may-20 |
| G51 | CGAB-Guatemalan Chamber of Food and Beverages | Facebook | Information management Coalition management | Amplification Fragmentation and destabilization | Esta semana presentaron en el Congreso una iniciativa de “Ley de Promoción de Alimentación Saludable”. La supuesta “buena intención” de la ley es que todos podamos tener una vida sana, pero en la práctica lo que hace es coartar nuestra libertad, imponiéndonos la visión de tecnócratas iluminados que desprecian la libertad. Desde ya declaro mi total oposición a todo este tipo de imposiciones, no solo porque amo y defiendo la libertad de todos, sino porque además, estas coacciones no funcionan. | <https://www.prensalibre.com/opinion/acabemos-con-los-obesos-matemos-a-los-desnutridos/?fbclid=IwAR0PIC4NieJ3zWyOzGPVKsgZaOlCXj46JbcqiGYYuIWS9nYdTx3p4Jh2dDA> | <https://www.facebook.com/Vida-Saludable-GT-228946467687201/> | 26-may-20 |
| G52 | CGAB-Guatemalan Chamber of Food and Beverages | Facebook | Information management | Amplification | De una manera velada, disfrazándolo de etiquetado, lo que esta iniciativa propone es ponerle un “sin tax” (impuesto al pecado) de hasta el 35 por ciento a los alimentos y bebidas “dañinos”, bajo la premisa paternalista de que las personas son tontas e ignorantes y hay que forzarlas a crear buenos hábitos, consumiendo productos que los reguladores consideran “saludables” y desincentivando que coman productos que los reguladores consideran “dañinos”. | <https://www.prensalibre.com/opinion/acabemos-con-los-obesos-matemos-a-los-desnutridos/?fbclid=IwAR0PIC4NieJ3zWyOzGPVKsgZaOlCXj46JbcqiGYYuIWS9nYdTx3p4Jh2dDA> | <https://www.facebook.com/Vida-Saludable-GT-228946467687201/> | 26-may-20 |
| G53 | CGAB-Guatemalan Chamber of Food and Beverages | Facebook | Discursive strategy  Information management Coalition management | Costs  Amplification Fragmentation and destabilization | El efecto principal de una legislación así es incrementar el costo de los productos que más consumen los pobres, con lo que se empeora su nivel de vida. Esto es especialmente importante en un país como Guatemala, donde la mitad de los niños padecen desnutrición crónica. ¿A quién en su sano juicio se le ocurre incrementar el precio de los alimentos en un país donde muchas personas viven en la pobreza y el principal problema es de desnutrición, no de obesidad? Los impulsores de esta iniciativa son conscientes de ese grave error de concepto, al grado de que intentan justificarla con el argumento de que “el 71% de los adultos en áreas urbanas padecen obesidad”, pero no mencionan a la mitad de los niños desnutridos en todo el país. ¡Mezquinos! | <https://www.prensalibre.com/opinion/acabemos-con-los-obesos-matemos-a-los-desnutridos/?fbclid=IwAR0PIC4NieJ3zWyOzGPVKsgZaOlCXj46JbcqiGYYuIWS9nYdTx3p4Jh2dDA> | <https://www.facebook.com/Vida-Saludable-GT-228946467687201/> | 26-may-20 |
| G54 | CGAB-Guatemalan Chamber of Food and Beverages | Facebook | Information Management Discursive strategy | Amplification Costs Contraband | Las consecuencias serán que, por un lado, se reducirá todavía más la industria de alimentos y bebidas en Guatemala, con los serios efectos que eso tendría sobre la economía de los guatemaltecos —menos empleo, menos actividad económica y hasta menos impuestos— y por el otro, aun considerando que fuera válida la teoría de la OMS —que no lo es—, de todos modos no se reduciría el consumo de los productos que ellos quieren que se reduzca porque estas regulaciones, por definición, no le aplicarían a los productos de contrabando. | <https://www.prensalibre.com/opinion/acabemos-con-los-obesos-matemos-a-los-desnutridos/?fbclid=IwAR0PIC4NieJ3zWyOzGPVKsgZaOlCXj46JbcqiGYYuIWS9nYdTx3p4Jh2dDA> | <https://www.facebook.com/Vida-Saludable-GT-228946467687201/> | 26-may-20 |
| G55 | CGAB-Guatemalan Chamber of Food and Beverages | Facebook | Discursive strategy Information Management | Frame the debate Amplification Suppression | Esta iniciativa propone regular un etiquetado frontal de advertencia sobre productos alimenticios utilizando sellos que pueden causar confusión al consumidor. Con más y mejor información, tomaremos mejores decisiones. Los sellos no ayudan a comparar, falta información para decidir. Las normativas internacionales vigentes promueven el uso de un etiquetado nutricional basado en datos cuantitativos que informan adecuadamente a las personas. Porque no siempre menos sellos es mejor #SellosPorPorcion #ComamosInformados |  | <https://www.facebook.com/228946467687201/videos/1920517671587947> | 26-may-20 |
| G56 | CGAB-Guatemalan Chamber of Food and Beverages | Facebook | Discursive strategy | Frame the debate | Desde la perspectiva de asociaciones empresariales de alimentos y bebidas de toda la región ALAIAB promueve la proactividad del sector privado para la creación de entornos laborales saludables y activos, la innovación permanente de la oferta alimentaria para la atención de necesidades nutricionales, tal es el caso de alimentos con menor contenido de sodio, azúcar y grasas; proporcionar información nutrimental, en apego a los lineamientos internacionales en materia de etiquetado; procurar la protección de la niñez, entre otras acciones que ya la industria alimentaria ejecuta en toda la región, alineada con los objetivos ratificados este 27 de septiembre por Naciones Unidas. |  | <https://www.facebook.com/photo/?fbid=311352232779957&set=pcb.311352276113286> | 26-may-20 |
| G57 | CGAB-Guatemalan Chamber of Food and Beverages | Facebook | Discursive strategy | Frame the debate | La Cámara Guatemalteca de Alimentos y Bebidas lanza la campaña iVida Saludable! con el objetivo de crear conciencia en las personas para adoptar un estilo de vida saludable. La -CGAB- desea brindar información objetiva y veraz con relación a los alimentos procesados, los que forman parte del balance nutricional diario de las personas en cantidad y calidad, facilitando la disponibilidad de alimentos. Los alimentos procesados se caracterizan por ser inocuos y seguros para el consumidor. |  | <https://www.facebook.com/photo/?fbid=229393400975841&set=a.229072481007933> | 26-may-20 |
| G58 | CGAB-Guatemalan Chamber of Food and Beverages | Facebook | Discursive strategy | Frame the debate | La CGAB y sus socios promueven y cumplen con la información nutricional de los productos y apoya el etiquetado que proporcione la información más clara y completa para que el consumidor tome su mejor decisión. |  | <https://www.facebook.com/photo/?fbid=2584989315159636&set=a.1909116992746875> | 26-may-20 |
| G59 | CGAB-Guatemalan Chamber of Food and Beverages | Facebook | Discursive strategy Information Management | Frame the debate Amplification | La CGAB comparte la mayoría de los criterios de la Dra. Socolovsky, el etiquetado nutricional debe cumplir con la función de informar adecuadamente al consumidor. Es importante que las personas se eduquen para que puedan interpretar lo que dice esa tabla.  El etiquetado de alimentos es una política pública , hay que tener presente que Guatemala es un país que tiene más desnutrición que obesidad, lo primero que tiene que atender es el hambre porque se ha comprobado que esos niños desnutridos de hoy serán los adolescentes obesos dentro de 10 años- De nada sirve la tabla si la gente no sabe lo que es una proteína o un ácido graso saturado. La educación nutricional es tan fundamental que debería empezar desde preprimaria. |  | <https://www.facebook.com/photo/?fbid=2584157428576158&set=a.1909116992746875> | 29-may-20 |
| G60 | CGAB-Guatemalan Chamber of Food and Beverages | Facebook | Discursive strategy | Costs | Al superar los límites de nutrientes, las empresas estarían obligadas a colocar tantos sellos de advertencia en sus etiquetas, como límites rebasados por nutriente. La forma de evitar el consumo “alto” de estos nutrientes es castigar con más costos a los productores formales de alimentos y bebidas con un nuevo impuesto cubierto en forma de una tasa de autorización de etiquetas, que va de 10% por un sello hasta 35% por los 5 sellos posibles. |  | <https://www.facebook.com/photo/?fbid=2232699323721972&set=a.1909116992746875> | 29-may-20 |
| G61 | CGAB-Guatemalan Chamber of Food and Beverages | Facebook | Information Management | Supression | Postura de la CGAB en torno a la iniciativa Ley de Promoción de Alimentación Saludable. La CGAB rechaza dicha iniciativa por considerar que no cuenta con fundamentos científicos y afectaría al consumidor. La iniciativa de ley de Promoción de Alimentación Saludable que busca que se coloque una advertencia nutricional en los productos con alto contenido de azúcar, grasas, sodio y edulcorantes, carece de fundamento científico, técnico económico según señaló la CGAB. De acuerdo con la Cámara, las instituciones de gobierno y organismos internacionales no pudieron explicar cómo se definieron los montos del nuevo impuesto y no contaban con estudios sobre el impacto en la economía familiar, las empresas fabricantes de alimentos y el precio de los productos. |  | <https://www.facebook.com/photo/?fbid=2246065015718736&set=a.1909116992746875> | 29-may-20 |
| G62 | CGAB-Guatemalan Chamber of Food and Beverages | Facebook | Information Management | Supression | Esta es la etiqueta que la ley vigente obliga a colocar en los productos y que contiene la información que requiere el consumidor.  ¿Por qué la iniciativa de Ley 5504 Ley de Promoción de Alimentación Saludable insiste en crear una etiqueta adicional, que no brinda nueva información al consumidor y por la cual se deberá pagar una nueva tasa impositiva de hasta 35% sobre el costo de producción? ¿Con esta nueva tasa impositiva los precios de los alimentos y bebidas aumentarán? |  | <https://www.facebook.com/photo/?fbid=2353980244927212&set=a.1909116992746875> | 29-may-20 |
| G63 | CGAB-Guatemalan Chamber of Food and Beverages | Facebook | Information Management | Supression | La Iniciativa 5504 Ley de Promoción de Alimentación Saludable propone sellos de advertencia nutricional que no dan información precisa de la cantidad de nutrientes que tiene el producto. Por lo que con esta iniciativa de ley se genera confusión al consumidor y lo engaña con argumentos de que gozará de una vida saludable (sin obesidad, sin diabetes, sin hipertensión, sin cáncer). Por eso, consideramos que esta iniciativa de ley no se debe aprobar y que las normas de etiquetado frontal se definan a través del Reglamento Técnico Centroamericano. Como se puede observar en el video, con la Iniciativa 5504 se repiten los mismos errores adoptados en otros países. |  | <https://www.facebook.com/cgab.org.gt/videos/553110435095955> | 29-may-20 |
| G64 | CGAB-Guatemalan Chamber of Food and Beverages | Facebook | Information Management | Supression | ¿Por qué no aprobar la iniciativa de Ley 5504 de Promoción y Alimentación Saludable? ¿Qué es la Iniciativa de ley? Una normativa sin fundamento técnico-científico sin investigación sustentada a nivel nacional No toma en cuenta la normativa internacional de CODEX Alimentarius Pretende normar los hábitos de consumo de alimentos y bebidas procesados de las personas sin darles mejor información Crea tasas impositivas Promueve el aumento de precios a los alimentos y bebidas procesados Establecer sellos de advertencia que confunden al consumidor porque no informan sobre el contenido y cantidad de los nutrientes de los alimentos y bebidas procesados Arbitrariamente define a qué alimentos y bebidas procesados se aplican los sellos de advertencia Riesgo por violación de acuerdos comerciales vinculantes para Guatemala Restringe la publicidad de alimentos y bebidas procesados, sin definir con claridad su objetivo  Puede violar derechos de propiedad intelectual |  | <https://www.facebook.com/photo/?fbid=2353238941668009&set=a.1909116992746875> | 29-may-20 |
| G65 | CGAB-Guatemalan Chamber of Food and Beverages | Facebook | Discursive strategy | Governance Costs | ¿Por qué no aprobar la iniciativa de Ley 5504 de Promoción y Alimentación Saludable? ¿Qué es la Iniciativa de ley? Limita la libre empresa porque pretende imponer el manejo de los costos y precios de los alimentos y bebidas procesadas Promueve el contrabando  Pone en riesgo puestos de trabajo y el comercio de alimentos y bebidas procesados |  | <https://www.facebook.com/photo/?fbid=2353238941668009&set=a.1909116992746875> | 29-may-20 |
| G66 | CGAB-Guatemalan Chamber of Food and Beverages | Facebook | Information management | Suppression | ¿Por qué no aprobar la iniciativa de Ley 5504 de Promoción y Alimentación Saludable? ¿Qué no es la Iniciativa de Ley? No es una solución a los gastos de salud pública por las enfermedades causadas por obesidad, hipertensión, diabetes, entre otras. No promueve el combate a la desnutrición No es un apoyo a la economía familiar porque aumenta los precios de la canasta básica No es una normativa que promueva hábitos de vida saludable en las personas No es una normativa integral para todos los alimentos que se consumen, únicamente pretende regular al 25 % de la canasta básica compuesta por alimentos y bebidas procesados No promueve los compromisos internacionales del país, exigiendo requisitos adicionales a los acordados con socios comerciales No promueve la calidad de los alimentos solo restringe el uso de ingredientes mal llamados críticos No aclara al consumidos la cantidad de ingredientes que debe consumir por día |  | <https://www.facebook.com/photo/?fbid=2353238941668009&set=a.1909116992746875> | 29-may-20 |
| G67 | CGAB-Guatemalan Chamber of Food and Beverages | Facebook | Information management | Amplification | Hoy se presentó al Congreso de la República la iniciativa de Ley de Promoción de Alimentación Saludable.  Esta iniciativa propone regular un etiquetado frontal de advertencia sobre productos alimenticios utilizando sellos que pueden causar confusión al consumidor.  Las normativas internacionales vigentes promueven el uso de un etiquetado nutricional basado en datos cuantitativos que informan adecuadamente a las personas.  Este video explica por qué la propuesta de ley no informará adecuadamente a las personas. |  | <https://www.facebook.com/cgab.org.gt/posts/2206760716315833> | 02-jun-20 |
| G68 | CGAB-Guatemalan Chamber of Food and Beverages | Facebook | Information management | Suppression | Te compartimos el siguiente video que habla sobre el etiquetado de los alimentos. https://m.youtube.com/watch?v=hCCpUf7wSA0  Los alimentos deben ser escogidos según el estado nutricional del consumidos, y el estado nutricional del consumidor obeso no es el mismo que el de un niño desnutrido por lo tanto hacer un etiquetado de advertencia por ejemplo el contenido de azúcar en una porción de alimento puede ser acorde a las necesidades de un paciente obeso o un consumidor obeso, pero no para un paciente desnutrido que necesita consumir tanto grasa como azúcar por lo que podría estar disuadiendo a una madre de comprar alimentos a sus hijos | <https://www.youtube.com/watch?v=hCCpUf7wSA0&fbclid=IwAR25P-IsYq1CtwGAtERBv0Rb84XqpYkVirUBXdcq091c259uad0yzU7n9Qs&app=desktop> | <https://www.facebook.com/cgab.org.gt/posts/2306224993036071> | 02-jun-20 |
| G69 | CGAB-Guatemalan Chamber of Food and Beverages | Twitter | Discursive strategy | Frame the debate | CGAB - Cámara Guatemalteca de Alimentos y Bebidas @cgab2018 La CGAB busca aclarar el acceso a la información nutricional que el consumidor necesita saber sobre la composición de los alimentos. |  | <https://twitter.com/cgab2018/status/1253030156207259651/photo/1> | 02-jun-20 |
| G70 | CGAB-Guatemalan Chamber of Food and Beverages | Twitter | Information management | Amplification | Hacer bien la compra tiene sus secretos y entre los más importantes está el de saber leer la etiqueta nutricional de los productos. ¿Cuánta grasa tiene este queso? ¿Lleva mucha azúcar aquel tomate frito? ¿Cuántas vitaminas hay en los yogures? Pero la mayoría de los compuestos químicos que contienen los alimentos no aparecen en la lista, aunque algunos influyen directamente en la calidad nutricional de los alimentos.  No hay una "etiqueta anti nutricional" pero los anti nutrientes existen y estos compuesto químicos dificultan que el organismo asimile los nutrientes de la dieta. |  | <https://twitter.com/cgab2018/status/1245008544660848643/photo/1> | 02-jun-20 |
| G71 | CGAB-Guatemalan Chamber of Food and Beverages | Twitter | Discursive strategy | Frame the debate | La CGAB comparte la mayoría de los criterios de la Dra. Socolovsky, el etiquetado nutricional debe cumplir con la función de informar adecuadamente al consumidor. El etiquetado nutricional en alimentos es una política pública. Hay que conseguir una alianza con la industria alimentaria y elaborar un programa con formas de alimentación población sobre como consumirlas. Hay que ser conscientes que si al inicio no se utiliza una campaña de educación es muy probable que s pierda el esfuerzo. |  | <https://twitter.com/cgab2018/status/1220086989497782273/photo/1> | 02-jun-20 |
| G72 | CGAB-Guatemalan Chamber of Food and Beverages | Twitter | Discursive strategy  Information management | Suppression | Como se puede observar en el video, con la Iniciativa 5504 (Ley de Alimentación Saludable) se repiten los mismos errores adoptados en otros países. Esta iniciativa de ley genera confusión al consumidor y lo engaña con argumentos de que gozará de una vida saludable sin obesidad, diabetes, hipertensión |  | <https://twitter.com/cgab2018/status/1126238602042707968> | 02-jun-20 |
| G73 | CGAB-Guatemalan Chamber of Food and Beverages | Twitter | Information management | Suppression | Este es el "tamal saludable" que la iniciativa de ley Promoción de Alimentación Saludable propone que comamos. Según recientes tendencias, el tamal que tradicionalmente comemos es responsable de obesidad, hipertensión, cáncer, diabetes, entre otras, por el alto contenido en sal y grasa.  El "tamal saludable" no le causará ninguna de estas enfermedades. |  | <https://twitter.com/cgab2018/status/1074713748693229568/photo/1> | 02-jun-20 |
| G74 | CGAB-Guatemalan Chamber of Food and Beverages | Twitter | Information management | Amplification | CGAB - Cámara Guatemalteca de Alimentos y Bebidas @cgab2018 Postura de la CGAB en torno a la iniciativa Ley de Promoción de Alimentación Saludable. El INCAP argumenta que la iniciativa pretende reducir el consumo de ciertos productos para disminuir enfermedades como el cáncer, la hipertensión, la diabetes y la obesidad. Cifras del Instituto señalan que el consumo de alimentos pre-envasados en la dieta del guatemalteco es de aproximadamente del 15%. Sobre este indicador, la CGAB sostiene que es un nivel modesto y que el proyecto no cuenta con un análisis científico que comprende la relación entre la legislación y la reducción de dichos padecimientos. Agregaron que la implementación de la ley incentivaría el contrabando, generando pérdidas económicas para las empresas y ocasionaría un aumento de precios de los productos afectando a los consumidores |  | <https://twitter.com/cgab2018/status/1069609180716302338/photo/1> | 02-jun-20 |
| G75 | CGAB-Guatemalan Chamber of Food and Beverages | Twitter | Information management | Amplification | CGAB - Cámara Guatemalteca de Alimentos y Bebidas @cgab2018 Comunicado de Prensa: Ley de Promoción de Alimentación Saludable será dictaminada por la Comisión de Salud del Congreso. (…) La propuesta de ley impone un nuevo impuesto a los alimentos con "altos" contenidos en azúcar, grasas, sodio y edulcorantes que puede representar entre 10% y 35% sobre el costo de producción de alimentos preparados. Se solicitó indicar el fundamento de estos números y al análisis del impacto sobre el consumo, resulto sorprendente que ninguna de las instituciones de gobierno y organismos internacionales pudiera explicar cómo se determinaron estas cifras y no contaban con estudios económicos sobre el impacto en la economía familiar y en el precio de los productos. INCAP aclaró que el objeto de estos impuestos es evitar el consumo de productos con estas características para disminuir enfermedades como el cáncer, la hipertensión, la diabetes y la obesidad, sin tomar en cuenta el derecho de los guatemaltecos al acceso a la alimentación. |  | <https://twitter.com/cgab2018/status/1068242847730737152/photo/2> | 02-jun-20 |
| G76 | Food and beverage trade-association of the Chamber of Industry of Guatemala | Facebook | Information management | Suppression | La afirmación de que los alimentos procesados "no son saludables" no encuentra respaldo en la ciencia 👨🏻‍🔬🔬. Al igual que los productos naturales, cada alimento procesado tiene determinados componentes y nutrientes que lo caracterizan. Como consumidor puedes definir tus opciones sin preocuparte si el alimento es procesado o no. Como lo hemos mencionado, los alimentos procesados son regulados por las autoridades correspondientes y son evaluados por organizaciones internacionales que afirman la seguridad de los mismos. ¡ Todo alimento es bueno siempre y cuando sea parte de una #DietaBalanceada ⚖🙋🏻‍♀️ ! #MartesDeMitos |  | <https://www.facebook.com/photo/?fbid=2181207242093531&set=pcb.2181207892093466> | 02-jun-20 |
| G77 | Food and beverage trade-association of the Chamber of Industry of Guatemala | Twitter | Information management | Amplification | Gremial de Alimentos y Bebidas retwitteó Codex Alimentarius @FAOWHOCodex Memorándum \| #Codex work on #nutrition and #labelling  Codex Alimentarius @FAOWHOCodex Rostro pensativo \| #DYK \| #Codex provides guidance on the compositional requirements of foods so that they are nutritionally safe. Codex also provides guidance on general labelling of #foods & the health or nutrient claims producers make on labels, with terms such as "low fat", "high fat" etc. |  | <https://twitter.com/FAOWHOCodex/status/1153561556438933504> | 02-jun-20 |
| G78 | Food and beverage trade-association of the Chamber of Industry of Guatemala | Twitter | Coalition management | Intern | Gremial de Alimentos y Bebidas retwitteó Sindicato de Industriales de Panamá @industrialespty Participando en la VI reunión de la Coalición de Alimentos y Bebidas de #centroamerica y en seguimiento a las acciones estratégicas en materia regulatoria de la región. #uniónaduanera #integración #entornoregulatorio #etiquetado  #nutrientes #alimentos  #bebidas #gremios |  | <https://twitter.com/industrialespty/status/1108511767943094273> | 10-jun-20 |
| G79 | Food and beverage trade-association of the Chamber of Industry of Guatemala | Twitter | Coalition management | Intern | Gremial de Alimentos y Bebidas retwitteó LiberalGt @GtLiberal En el marco del 1er. Foro de la Industria de Alimentos y Bebidas: Desafíos de la agenda regional, se lleva a cabo el Panel “la importancia del etiquetado y armonización en Centroamérica” con distintos expertos en la temática.  @industriaguate |  | <https://twitter.com/GtLiberal/status/1108844080539361280> | 10-jun-20 |
| G80 | Food and beverage trade-association of the Chamber of Industry of Guatemala | Twitter | Information management | Amplification | Gremial de Alimentos y Bebidas retwitteó Cámara de Industria de Guatemala @industriaguate “Una política de promoción de la salud debe avanzar por la vía de fortalecimiento de los modelos informativos y no de advertencia. En el caso de #Centroamérica, con la aplicación regional” indica Mario Montero |  | <https://twitter.com/industriaguate/status/1108775529916915715> | 10-jun-20 |
| G81 | ALAIAB- Latin American Alliance of Food and Beverage Industry Associations | Website | Discursive strategy  Information management | Frame the debate  Amplification | ALAIAB presenta sus observaciones ante el informe de la Comisión de Alto Nivel de la OMS contra las ENTs​. (...)Desde la perspectiva de asociaciones empresariales de alimentos y bebidas de toda la región, ALAIAB promueve la proactividad del sector privado para la creación de entornos laborales saludables y activos; la innovación permanentemente de la oferta alimentaria para la atención de necesidades nutricionales, tal es el caso de alimentos con menor contenido de sodio, azúcares y grasas; proporcionar información nutrimental, en apego a los lineamientos internacionales en materia de etiquetado; procurar la protección de la niñez, entre otras acciones que ya la industria alimentaria ejecuta en toda la región, alineadas con los objetivos ratificados este 27 de setiembre por Naciones Unidas. |  | <https://alaiab.org/wp/alaiab-presenta-observaciones-ante-informe-de-la-oms/> | 10-jun-20 |
| G82 | ALAIAB- Latin American Alliance of Food and Beverage Industry Associations | Website | Discursive strategy  Information management | Frame the debate  Amplification | LATAM: ALAIAB define sus recomendaciones para la construcción de marcos normativos de etiquetado frontal nutricional en la región. Por tratarse de asociaciones y empresas fabricantes de alimentos y bebidas, compartimos la importancia de incidir positivamente sobre la salud de la población a través de una visión unificada en toda la región, promovida por una red de organizaciones y empresas que comparten una serie de ejes estratégicos como: a. el desarrollo de una oferta de productos que innova permanentemente, capaz de atender los diversos requerimientos de alimentación y nutrición de la sociedad moderna; b. La utilización de sistemas de información al consumidor que promuevan la lectura del etiquetado nutricional y de esta forma, se incentiven las decisiones de ingesta apropiada y balanceada; c. la aplicación de las mejores prácticas empresariales en materia de publicidad y marketing, especialmente cuando la comunicación de las empresas fabricantes de alimentos y bebidas es recibida por públicos menores de edad, y d. la promoción de las mejores prácticas empresariales, institucionales y de política público-privada, en materia de educación y práctica de estilos de vida activos y saludables, en el ámbito internacional. |  | <https://alaiab.org/wp/alaiab-define-sus-recomendaciones-para-la-construccion-de-marcos-normativos-de-etiquetado-frontal-nutricional/> | 10-jun-20 |
| G83 | ALAIAB- Latin American Alliance of Food and Beverage Industry Associations | Website | Discursive strategy | Frame the debate | LATAM: ALAIAB define sus recomendaciones para la construcción de marcos normativos de etiquetado frontal nutricional en la región. (...)Un pilar importante para la promoción de estilos de vida saludable, son las acciones que desde la empresa privada se pueden realizar, para informar y educar al consumidor sobre el contenido de nutrientes en los productos. Para tales efectos, el etiquetado nutricional es una herramienta que incentiva decisiones de ingesta balanceadas |  | <https://alaiab.org/wp/alaiab-define-sus-recomendaciones-para-la-construccion-de-marcos-normativos-de-etiquetado-frontal-nutricional/> | 10-jun-20 |
| G84 | ALAIAB- Latin American Alliance of Food and Beverage Industry Associations | Website | Discursive strategy | Frame the debate | LATAM: ALAIAB define sus recomendaciones para la construcción de marcos normativos de etiquetado frontal nutricional en la región (...)De ahí que ALAIAB concuerda con todas aquellas voluntades públicas y privadas que buscan implementar sistemas de etiquetado nutricional que logren informar al consumidor y que incentiven la lectura sobre los nutrientes contenidos en los alimentos, como un hábito saludable. En este sentido, seremos aliados de los programas y políticas que busquen brindar a los consumidores la información sobre los alimentos, necesaria para mantener una dieta saludable. |  | <https://alaiab.org/wp/alaiab-define-sus-recomendaciones-para-la-construccion-de-marcos-normativos-de-etiquetado-frontal-nutricional/> | 10-jun-20 |
| G85 | ALAIAB- Latin American Alliance of Food and Beverage Industry Associations | Website | Information management | Amplification | LATAM: ALAIAB define sus recomendaciones para la construcción de marcos normativos de etiquetado frontal nutricional en la región (...)Por nuestro carácter de entidad regional, compuesta por organizaciones empresariales de todo el hemisferio, en ALAIAB estamos comprometidos con la promoción de marcos normativos de etiquetado general y/o nutricional, cuya efectividad se pueda garantizar por el cumplimiento de las siguientes características: 1. Generan información nutricional veraz, basada en evidencia científica robusta y en hechos. 2. Presenta la información de manera sencilla, promoviendo el papel del alimento en una dieta equilibrada, diversificada e inclusiva. 3. Se aplica a todos los grupos alimentarios, para facilitar la comparabilidad de las características nutricionales entre alimentos específicos o entre grupos alimentarios, como factor importante para las decisiones de ingesta integral balanceada. 4. De fácil aplicación e interpretación, que no genere error, confusión o engaño al consumidor o le induzca a pensar que un alimento o sus nutrientes, por sí mismos, tiene propiedades, características o efectos no demostrados, o en su defecto, que pueden ser dañinos para su salud. 5. Empodera al consumidor para la toma de decisiones autónomas, dentro de un contexto de estilo de vida saludable. 6. Consistencia con lineamientos generales del CODEX ALIMENTARIUS 7. Con base en los lineamientos del CODEX ALIMENTARIUS, eviten la utilización de advertencias, ilustraciones o signos gráficos que pueden generar prejuicios subjetivos sobre la pertinencia nutricional de productos o sus nutrientes. 8. Su base científica facilite la armonización y convergencia normativa entre países para garantizar el debido equilibrio entre la promoción de la salud y la fluidez del comercio. |  | <https://alaiab.org/wp/alaiab-define-sus-recomendaciones-para-la-construccion-de-marcos-normativos-de-etiquetado-frontal-nutricional/> | 10-jun-20 |
| G86 | ALAIAB- Latin American Alliance of Food and Beverage Industry Associations | Website | Discursive strategy | Good governance | LATAM: ALAIAB define sus recomendaciones para la construcción de marcos normativos de etiquetado frontal nutricional en la región. (...)Transparencia, diálogo y consulta inclusiva: La elaboración de marcos normativos en materia de etiquetado de alimentos debe ser transparente y consultativo. Promovemos que en todos los países se aplique y respete la institucionalidad relativa a los procesos de construcción de marcos normativos y reglamentos técnicos, donde la participación abierta, participativa e incluyente se encuentra tutelada por los procedimientos y normativas ya establecidas por los países. Los procesos de construcción de la normativa deben ser producto de un intercambio que involucra transversalmente a todos los actores, incluyendo a los puntos focales de los ministerios que atiendan asuntos del agro, la industria, la producción y el comercio, para considerar todas las implicancias y los impactos que esto trae aparejado en la producción, el comercio y el consumo. |  | <https://alaiab.org/wp/alaiab-define-sus-recomendaciones-para-la-construccion-de-marcos-normativos-de-etiquetado-frontal-nutricional/> | 10-jun-20 |
| G87 | ALAIAB- Latin American Alliance of Food and Beverage Industry Associations | Website | Discursive strategy | Good governance | LATAM: ALAIAB define sus recomendaciones para la construcción de marcos normativos de etiquetado frontal nutricional en la región. (...)Respeto al marco institucional pertinente: La institucionalidad pertinente e históricamente utilizada para estos efectos, es el CODEX ALIMENTARIUS. Es CODEX el que asegura la construcción de marcos normativos de referencia, que cuentan con el sustento científico necesario y además el que aporta otras características de alta conveniencia para gobiernos y operadores del mercado, tales como:  i. Cuenta con la participación inclusiva de gobiernos, academia, consumidores, empresa privada, entre otros. ii. Resguarda los principios básicos de evidencia científica en procura de razonabilidad técnica, equilibrio, credibilidad y efectividad. iii. Procura la armonización normativa internacional y el respeto de los países a sus compromisos comerciales internacionales, minimizando los obstáculos técnicos al comercio y las afectaciones innecesarias a la inversión. |  | <https://alaiab.org/wp/alaiab-define-sus-recomendaciones-para-la-construccion-de-marcos-normativos-de-etiquetado-frontal-nutricional/> | 15-jun-20 |
| G88 | ALAIAB- Latin American Alliance of Food and Beverage Industry Associations | Website | Discursive strategy | Good governance | LATAM: ALAIAB define sus recomendaciones para la construcción de marcos normativos de etiquetado frontal nutricional en la región. (...)Marco de cooperación público privado para la educación. El trabajo conjunto y de colaboración público privada, debe arrojar también procesos de educación al consumidor para el efectivo uso y aprovechamiento de la información nutricional de los alimentos por parte de los consumidores. |  | <https://alaiab.org/wp/alaiab-define-sus-recomendaciones-para-la-construccion-de-marcos-normativos-de-etiquetado-frontal-nutricional/> | 15-jun-20 |
| G89 | ALAIAB- Latin American Alliance of Food and Beverage Industry Associations | Website | Discursive strategy | Good governance | ​​Tanto los representantes de ALAIAB como el señor Da Costa, coincidieron en la necesidad de trabajar para el fortalecimiento de los principios sobre los que se basa el CODEX ALIMENTARIUS: solidez científica de los procesos normativos y regulatorios que se desarrollan, así como la importancia de evitar obstáculos técnicos al comercio innecesarios.​​  Dicha conclusión se genera a partir de la inquietud planteada por las asociaciones de alimentos del hemisferio, referente a la ruta unilateral que algunos países están siguiendo en temas que tradicionalmente han estado bajo la tutela del CODEX, tal es el caso del etiquetado nutricional. Sobre este tema en particular, el sector privado ha identificado un marcado alejamiento de algunos países de las directrices generales del CODEX, llevando algunas posturas regulatorias a alejarse del concepto básico de fundamentación y evidencia científica, provocándose un preocupante proceso de desarmonización entre los marcos normativos de los países, tema que fue de amplia reflexión por parte del grupo. |  | <https://alaiab.org/wp/industria-alimentaria-de-latam-se-reune-con-presidente-mundial-del-codex/> | 15-jun-20 |
| G90 | ALAIAB- Latin American Alliance of Food and Beverage Industry Associations | Website | Discursive strategy | Good governance | (...) Diversas iniciativas regulatorias que buscan modificar la reglamentación vigente en la región en materia de etiquetado nutricional. Se puso atención a la propuesta recientemente presentada por el Consejo de Ministros de Salud de la región al Consejo de Ministros de Economía que busca la implementación de un modelo de etiquetado de advertencia en los alimentos y bebidas procesados. |  | <https://www.cacia.org/coalicion-centroamericana-de-alimentos-y-bebidas-analiza-las-tendencias-del-entorno-regulatorio-en-la-region/> | 15-jun-20 |
| G91 | CACIA- Central American Coalition of the Food and Beverage Industry | Website | Coalition management | Intern | La Cámara, en defensa de sus intereses, participa y lidera en diferentes comités que le brindan soporte al MEIC en la búsqueda de posturas país que sean equilibradas y justas para la industria alimentaria nacional, de manera tal que los acuerdos en los foros mundiales, donde se generan normativas que afectan al sector tengan además de una validez científica, un enfoque que no genere obstáculos para el sano desarrollo de la industria local.  Algunos de los comités activos son:  Nutrición y Alimentos para Regímenes Especiales. Aditivos Alimentarios. Etiquetado. |  | <https://www.cacia.org/areas-estrategicas/regulacion-alimentaria/> | 15-jun-20 |
| G92 | CACIA- Central American Coalition of the Food and Beverage Industry | Website | Coalition management | Intern | Comisión Técnica Consultiva del Valor Nutritivo de los Alimentos Esta comisión de trabajo de la que CACIA(Cámara Costarricense de la Industria Alimentaria) tiene como objetivo coordinar y promover acciones relacionadas con el adecuado uso de la información sobre el valor nutritivo de los alimentos, es decir todo lo relacionado con etiquetado nutricional. Esta comisión intersectorial está conformada por profesionales en la materia y por las instituciones rectoras del estado. Para ello posee un procedimiento de atención de consultas en etiquetado nutricional. Producto de estas consultas, solicita a los productores y comercializadores que de forma voluntaria realicen las correcciones pertinentes con tal de que el consumidor reciba la información nutricional correcta. |  | <https://www.cacia.org/areas-estrategicas/regulacion-alimentaria/> | 15-jun-20 |
| G93 | CACIA- Central American Coalition of the Food and Beverage Industry | Website | Direct involvement and influence in policy | Actors in government decision making | Brasil y México someten a consulta pública propuestas regulatorias de etiquetado frontal de advertencia. CACIA reacciona. Debido a la sensibilidad que este tipo de medidas implica para una industria como la nuestra y por la clara violación a los principios básicos de la normativa internacional tutelada por el CODEX ALIMENTARIUS, así como la violación que este tipo de medidas implica para acuerdos y compromisos internacionales que los países han adoptado, CACIA solicitó al Ministerio de Comercio Exterior la debida e inmediata participación de Costa Rica en este proceso de consulta, con miras a manifestar el impacto de la medida, así como la creación de nuevas barreras al comercio que impedirá, aún más, el ingreso de nuestros productos a ese mercado. Igualmente, CACIA de manera directa ha enviado sus observaciones al gobierno mexicano, con todas sus observaciones y descripciones sobre todos los principios técnicos, jurídicos y científicos que una medida de este tipo violenta. |  | <http://www.cacia.org/brasil-y-mexico-someten-a-consulta-publica-propuestas-regulatorias-de-etiquetado-frontal-de-advertencia-cacia-reacciona/> | 15-jun-20 |
| G94 | CACIA- Central American Coalition of the Food and Beverage Industry | Website | Direct involvement and influence in policy | Actors in government decision making | (...) Adicionalmente, a partir de la discusión internacional en materia de etiquetados de advertencia y las propuestas hechas por Costa Rica (etiquetado nutricional frontal basado en las GDA´s) y por el Consejo de Ministros de Salud de la región (etiquetado de advertencia según modelo chileno basado en el perfil de nutrientes de OPS), los países también acordaron iniciar un proceso de revisión de la materia. Sobre este particular no hay una visión unificada entre los países.  No obstante lo anterior, se espera que primero suceda el proceso de actualización del RTCA de Etiquetado Nutricional. Por tal motivo, actualmente CACIA trabaja en coordinación con el Consejo Consultivo Empresarial de la Integración Económica (CCIE), y con el apoyo de un grupo importante de federaciones de productores del sector privado, en el análisis de la temática, de manera tal que se garantice la participación abierta, transparente y consultativa del sector privado en este proceso. |  | <http://www.cacia.org/cacia-se-reune-con-ministras-de-economia-y-comercio-exterior-codex-y-etiquetados-de-advertencia-llenan-agendas/> | 15-jun-20 |
| G95 | CACIA- Central American Coalition of the Food and Beverage Industry | Website | Direct involvement and influence in policy  Information management | Actors in government decision making  Amplification | Durante el último trimestre de 2019, CACIA desarrolló una intensa labor de diálogo con las Ministras Victoria Hernández de Economía, Industria y Comercio, así como Dyalá Jiménez, de la cartera de Comercio Exterior. El objetivo principal buscado y logrado fue el apoyo de ambas autoridades a temas trascendentales para la industria alimentaria: una participación decidida de Costa Rica en los foros mundiales del CODEX ALIMENTARIUS, en los que actualmente el país lidera y preside comités importantes, tal es el caso del comité que desarrolla los temas relativos a los etiquetados nutricionales, así como la normativa en materia de regímenes especiales, entre otros. Igualmente es determinante la consolidación de una postura país que promueva normativas regionales de etiquetado nutricional, respetuosas de los lineamientos del CODEX, así como de los principios básicos de evidencia científica y evasión de obstáculos técnicos al comercio innecesario. |  | <http://www.cacia.org/cacia-se-reune-con-ministras-de-economia-y-comercio-exterior-codex-y-etiquetados-de-advertencia-llenan-agendas/> | 15-jun-20 |
| G96 | CACIA- Central American Coalition of the Food and Beverage Industry | Website | Direct involvement and influence in policy  Information management | Actors in government decision making  Supression | Con ambos ministerios CACIA ha promovido la necesidad de que la región tenga marcos normativos de etiquetado nutricional que cumplan una serie de principios, que sean informativos y eduquen a la población en materia de buenos hábitos de alimentación; no obstante, en la actualidad florecen iniciativas que buscan implementar modelos gráficos de advertencia, los cuales no cuentan con la debida justificación que valide la necesidad de una medida tan extrema en los alimentos industrializados. |  | <http://www.cacia.org/cacia-se-reune-con-ministras-de-economia-y-comercio-exterior-codex-y-etiquetados-de-advertencia-llenan-agendas/> | 15-jun-20 |
| G97 | CACIA- Central American Coalition of the Food and Beverage Industry | Website | Information management | Suppresion | CACIA expone sus preocupaciones frente a gobiernos por proliferación de etiquetados de advertencia en América Latina. Los etiquetados de advertencia que dañan innecesariamente la imagen de los alimentos procesados siguen en aumento en nuestra región. Ya tenemos modelos de advertencia implementados en Ecuador, Bolivia, Uruguay, Chile, Perú y actualmente pasando por los procesos de consulta pública interna y consulta internacional ante OMC en Brasil y México respectivamente |  | <http://www.cacia.org/cacia-expone-sus-preocupaciones-frente-a-gobiernos-por-proliferacion-de-etiquetados-de-advertencia-en-america-latina/> | 15-jun-20 |
| G98 | CACIA- Central American Coalition of the Food and Beverage Industry | Website | Direct involvement and influence in policy  Information management | Actors in government decision making  Supression | CACIA expone sus preocupaciones frente a gobiernos por proliferación de etiquetados de advertencia en América Latina.  CACIA ha enviado comentarios manifestando sus preocupaciones ante este tipo de modelos gráficos por carecer de sustento científico, ya que desde nuestra perspectiva, no existe ningún alimentos, que por sus características particulares, tipos de procesamiento, ingredientes o nutrientes que utilice, tiene el potencial de generar enfermedades particulares. |  | <http://www.cacia.org/cacia-expone-sus-preocupaciones-frente-a-gobiernos-por-proliferacion-de-etiquetados-de-advertencia-en-america-latina/> | 17-jun-20 |
| G99 | CACIA- Central American Coalition of the Food and Beverage Industry | Website | Information management | Suppresion | CACIA expone sus preocupaciones frente a gobiernos por proliferación de etiquetados de advertencia en América Latina (...)Todas estas propuestas de etiquetado, se están desarrollando en diversos países a partir de procesos políticos y debates más ideológicos que políticos, pues parten de la premisa de que el incremento en la obesidad y el sobre peso de la población, se debe al incremento en las ventas de alimentos industrializados. |  | <http://www.cacia.org/cacia-expone-sus-preocupaciones-frente-a-gobiernos-por-proliferacion-de-etiquetados-de-advertencia-en-america-latina/> | 17-jun-20 |
| G100 | CACIA- Central American Coalition of the Food and Beverage Industry | Website | Information management | Suppresion | Que un alimento sea catalogado como “bueno” a partir de estos perfiles nutricionales, no significa automáticamente que la ingesta total individual será balanceada y adecuada en tiempos, porciones y demás factores que construyen una alimentación saludable. Es decir, el perfil nutricional de “alimento perfecto” establecido para un producto o grupo específico, no determina las demás variables que caracterizan una alimentación saludable |  | <http://www.cacia.org/cacia-expone-sus-preocupaciones-frente-a-gobiernos-por-proliferacion-de-etiquetados-de-advertencia-en-america-latina/> | 17-jun-20 |
| G101 | CACIA- Central American Coalition of the Food and Beverage Industry | Website | Discursive strategy | Good governance | Cuando esta tendencia inició, CACIA solicitó a nuestro Ministerio de Comercio Exterior (COMEX) presentar la preocupación comercial en el Comité de Obstáculos Técnicos al Comercio en Ginebra. Junto a Costa Rica, Guatemala y Estados Unidos también presentaron las mismas preocupaciones contra el etiquetado de advertencia de Chile y Ecuador.  Por las razones anteriores, en días recientes hemos insistido frente a nuestras autoridades, por la necesidad de velar porque los procesos normativos de alimentos a nivel internacional, respeten los principios fundamentales de seguridad jurídica, respeto a la institucionalidad, apego a las directrices del CODEX ALIMENTARIUS, armonización normativa internacional, apego a la ciencia y evasión de obstáculos innecesarios al comercio, todos valores debidamente tutelados por los compromisos y tratados internacionales que todos los países han firmado. Para un país pequeño, abierto al comercio internacional y con economías de escala de país pequeño, el irrespeto de los países a los compromisos vigentes en esta materia, genera una seria amenaza de quedar fuera de los mercados internacionales. |  | <http://www.cacia.org/cacia-expone-sus-preocupaciones-frente-a-gobiernos-por-proliferacion-de-etiquetados-de-advertencia-en-america-latina/> | 17-jun-20 |
| G102 | CACIA- Central American Coalition of the Food and Beverage Industry | Website | Information management | Suppresion | Esta metodología (etiquetado frontal) ha preocupado mucho a la industria alimentaria a nivel mundial, por carecer de fundamento técnico. Países como México, Chile, Perú, Ecuador, Uruguay y Brasil, la están utilizando para definir esos “puntos de corte” para calificar alimentos, generándose además una importante desarmonización regulatoria, por lo que Costa Rica hace dos años alzó la voz para que los países se pongan de acuerdo y establezcan lineamientos generales para definir los perfiles nutricionales. |  | <http://www.cacia.org/cacia-representara-a-la-industria-alimentaria-nacional-en-reunion-mundial-de-etiquetado-nutricional-en-alemania/> | 17-jun-20 |
| G103 | CACIA- Central American Coalition of the Food and Beverage Industry | Website | Discursive strategy | Good governance | Es en el foro del Codex Alimentarios donde se elaboran las normas y directrices que deben de cumplir los alimentos envasados en todos los países y donde se exponen los temas de mayor preocupación que deben ser atendidos, una de las prioridades es este tema del etiquetado frontal, el cual se espera que esté listo para el año 2021. De esta manera, los consumidores podrán comprender mejor la información sobre los alimentos que compran en cualquier parte del mundo y tomar mejores decisiones sobre su alimentación. |  | <http://www.cacia.org/codex-alimentarius-participamos-en-la-reunion-mundial-de-etiquetado-de-alimentos-en-canada/> | 17-jun-20 |
| G104 | CACIA- Central American Coalition of the Food and Beverage Industry | Website | Information management Discursirve strategy | Supression Frame the debate | Para CACIA es fundamental que toda norma que se elabore a nivel internacional o a nivel nacional cuente con el sustento técnico que la respalde, no en criterios subjetivos o intereses políticos, CACIA siempre defiende en los diferentes foros de discusión técnica que cualquier directriz que atienda las necesidades de la población, se base en los estudios de los expertos, así hemos logrado apoyar políticas públicas y trabajar en conjunto sector público-sector privado en programas exitosos como el de fortificación de alimentos y el programa nacional de reducción de sodio, entre otros, y esperamos seguir desarrollando nuevos proyectos, como la mejora de la presentación de la información al consumidor a través del etiquetado en general |  | <http://www.cacia.org/codex-alimentarius-participamos-en-la-reunion-mundial-de-etiquetado-de-alimentos-en-canada/> | 17-jun-20 |
| G105 | CACIA- Central American Coalition of the Food and Beverage Industry | Website | Direct involvement and influence in policy | Development and promotion of public-private initiatives and self-regulation | Sobre esta materia, actualmente se analizan dos propuestas de reforma a dicha normativa: una propuesta por el gobierno de Costa Rica, que plantea evolucionar la normativa actual, hacia un etiquetado nutricional en la parte frontal de los alimentos y las bebidas, que formaliza la metodología de las Guías Diarias de Alimentación, que hoy día son de amplia utilización de manera voluntaria por buena parte de la industria formal de la región. |  | <http://www.cacia.org/coalicion-centroamericana-de-alimentos-y-bebidas-da-seguimiento-a-entorno-regulatorio-poco-amigable/> | 17-jun-20 |
| G106 | CACIA- Central American Coalition of the Food and Beverage Industry | Website | Coalition management | Intern | El pasado mes de marzo, se reunió en ciudad Guatemala la Coalición Centroamericana de Asociaciones y Empresas de la Industria Alimentaria y de Bebidas (...)Uno de los principales resultados de esta reunión, es que las asociaciones de todos los países han acordado manifestar ante las autoridades de todos los países, una postura positiva y propositiva en materia de promoción de la salud a través del etiquetado nutricional informativo. El grupo de asociaciones representativas de la industria de alimentos y bebidas miran de manera positiva que se genere un proceso de revisión de la normativa centroamericana de etiquetado nutricional, de manera tal que se facilite la lectura de la información de los alimentos y así se genere un mayor empoderamiento del consumidor, para que tome las mejores decisiones de ingesta alimentaria, según las necesidades nutrimentales que son propias de las condiciones particulares de cada individuo. |  | <http://www.cacia.org/coalicion-centroamericana-de-alimentos-y-bebidas-da-seguimiento-a-entorno-regulatorio-poco-amigable/> | 17-jun-20 |
| G107 | CACIA- Central American Coalition of the Food and Beverage Industry | Website | Direct involvement and influence in policy | Direct access-lobbying Development and promotion of public-private initiatives and self-regulation | En esta ocasión, las organizaciones industriales representativas de la industria de alimentos y bebidas, manifestaron su preocupación ante enfoques regulatorios orientados a provocar una reacción negativa del consumidor frente a la decisión de compra de los productos. En una misiva enviada a los jerarcas de Comercio, Economía y Salud, los empresarios manifiestan la positiva y proactiva posición del sector, por avanzar en programas y políticas tendientes a la promoción de la salud, mediante el estímulo de los estilos de vida saludable, la innovación en la oferta para la reducción de azúcares, grasas y sodio, la promoción de las buenas prácticas empresariales en materia de publicidad de alimentos, el permanente apoyo a las políticas de fortificación de alimentos o la implementación de esquemas de etiquetado que estimulen la ingesta alimentaria informada y educada, entre otras iniciativas. |  | <http://www.cacia.org/4734-2/> | 17-jun-20 |
| G108 | CACIA- Central American Coalition of the Food and Beverage Industry | Website | Direct involvement and influence in policy | Actors in government decision making | Por tal motivo y ante la consulta interinstitucional, la Ministra de Salud ha respondido oficialmente que la posición de su Ministerio a cargo sigue manteniendo la misma coherencia nacional. Explica que en el momento que la propuesta fue puesta a conocimiento por parte del INCAP en el foro de COMISCA, la posición de Costa Rica fue la de someter dichas propuestas al escrutinio técnico de las instancias nacionales respectivas, motivo por la que Costa Rica no apoya el enfoque normativo de etiquetado frontal de advertencia propuesto por INCAP/COMISCA. |  | <http://www.cacia.org/ministra-de-salud-de-costa-rica-aclara-posicion-pais-sobre-etiquetado-frontal-de-advertencia/> | 17-jun-20 |
| G109 | CACIA- Central American Coalition of the Food and Beverage Industry | Website | Discursive strategy | Costs | Las propuestas tienden a reglamentar el etiquetado nutricional en el frente del envase de los alimentos y bebidas pre envasados, el cual es de especial sensibilidad para este sector productivo, que en el ámbito de nuestra región es responsable del 27% del Intercambio Intracentroamericano.  Para CACIA el enfoque extremista de regulaciones como las propuestas, ponen en peligro los más de 293.000 puestos de empleo que este sector productivo genera solamente en el ámbito industrial, y sobre todo, el enorme estímulo a la informalidad, en una industria conformada por más de 90% por la micro y pequeña empresa centroamericana. |  | <http://www.cacia.org/ministra-de-salud-de-c-r-apoya-resolucion-de-ministros-de-la-region-que-propone-etiquetado-de-prevencion-cacia-protesta-energicamente-ante-poder-ejecutivo/> | 17-jun-20 |
| G110 | CACIA- Central American Coalition of the Food and Beverage Industry | Website | Coalition management | Intern | El principal objetivo de la participación de CACIA era lograr la continuidad de un documento que se trabajó durante el año pasado y que fue liderado por Costa Rica y Nueva Zelanda para desarrollar lineamientos generales para etiquetado frontal. En este sentido se logró que este trabajo siga siendo liderado por ambos países.  El documento va a incluir qué es el etiquetado frontal y qué aspectos deben o no ser considerados para desarrollar un esquema, en caso de que un país desee implementarlo como parte de su estrategia para combatir el sobrepeso, la obesidad y las enfermedades crónicas no transmisibles. |  | <http://www.cacia.org/cacia-ante-el-comite-del-codex-en-paraguay-el-mundo-requiere-lineamientos-de-etiquetado-frontal-basado-en-ciencia/> | 17-jun-20 |
| G111 | CACIA- Central American Coalition of the Food and Beverage Industry | Website | Coalition management | Intern | Otro de los objetivos logrados durante la reunión fue que el Comité de Etiquetado aprobara consultarle al Comité de Nutrición si es pertinente realizar trabajos sobre perfiles nutricionales para complementar los lineamientos de etiquetado frontal.  Los perfiles nutricionales ya se utilizan en varios países, sin embargo, existe una alta desarmonización en sus contenidos, por lo que Costa Rica elevó la voz para llamar la atención en este sentido y buscar elementos de armonización que faciliten el comercio y que además tengan bases científicas claras. |  | <http://www.cacia.org/cacia-ante-el-comite-del-codex-en-paraguay-el-mundo-requiere-lineamientos-de-etiquetado-frontal-basado-en-ciencia/> | 17-jun-20 |
| G112 | CACIA- Central American Coalition of the Food and Beverage Industry | Website | Information management | Amplification | Los indicadores de impacto un año después de implementado este esquema, se evidenció que más del 80% de los consumidores lo conocen, consideran que es fácil de entender, que contiene información relevante y quisieran verlo en más productos.  En América las Guías Diarias de Alimentación han sido ampliamente difundidas por las asociaciones de fabricantes y por las empresas de alimentos y bebidas en muchos países. En este sentido, vemos iniciativas importantes en Estados Unidos, México, Costa Rica, Chile, Colombia, Argentina, entre otros, donde no solamente la industria los aplica, sino que existen programas de difusión y educación al consumidor, orientados a la promoción de una alimentación informada y balanceada que ayuda a mantener un estilo de vida saludable. |  | <http://estilosdevida.cacia.org/las-guias-diarias-alimentacion/> | 17-jun-20 |
| G113 | CACIA- Central American Coalition of the Food and Beverage Industry | Website | Information management | Amplification | Las Guías Diarias de Alimentación (GDA’s) son gráficas muy sencillas que nos informan sobre el contenido de calorías, grasas, azúcares y sodio presentes en los alimentos que consumimos. Han sido desarrolladas mundialmente por las industrias alimentarias como mecanismo para educar e informar a los consumidores, de manera tal que estos tengan mejores elementos para decisiones de consumo mucho más adecuadas y balanceadas, acorde a sus realidades personales. Tienen la enorme ventaja de que son de fácil lectura y ubicación. Su diseño está pensado para que las personas lo ubiquen fácilmente y para que su comprensión sea sencilla e invite a una lectura más llamativa, fresca y fácil de entender. |  | <http://estilosdevida.cacia.org/las-guias-diarias-alimentacion/> | 17-jun-20 |
